# METTL1 promotes tumorigenesis through tRNA-derived fragment biogenesis in prostate cancer

**DOI:** 10.1186/s12943-023-01809-8

**Published:** 2023-07-29

**Authors:** Raquel García-Vílchez, Ana M. Añazco-Guenkova, Sabine Dietmann, Judith López, Virginia Morón-Calvente, Silvia D’Ambrosi, Paz Nombela, Kepa Zamacola, Isabel Mendizabal, Saioa García-Longarte, Amaia Zabala-Letona, Ianire Astobiza, Sonia Fernández, Alejandro Paniagua, Borja Miguel-López, Virginie Marchand, Diego Alonso-López, Angelika Merkel, Ignacio García-Tuñón, Aitziber Ugalde-Olano, Ana Loizaga-Iriarte, Isabel Lacasa-Viscasillas, Miguel Unda, Mikel Azkargorta, Félix Elortza, Laura Bárcena, Monika Gonzalez-Lopez, Ana M. Aransay, Tomás Di Domenico, Manuel A. Sánchez-Martín, Javier De Las Rivas, Sònia Guil, Yuri Motorin, Mark Helm, Pier Paolo Pandolfi, Arkaitz Carracedo, Sandra Blanco

**Affiliations:** 1 Centro de Investigación del Cáncer and Instituto de Biología Molecular y Celular del Cáncer, Consejo Superior de Investigaciones Científicas (CSIC)-University of Salamanca, 37007 Salamanca, Spain; 2 Instituto de Investigación Biomédica de Salamanca (IBSAL), Hospital Universitario de Salamanca, 37007 Salamanca, Spain; 3 Washington University School of Medicine in St. Louis, 660S. Euclid Ave, St. Louis, MO 63110 USA; 4 Present Address: Department of Neurosurgery, Cancer Center Amsterdam, Amsterdam UMC, Vrije Universiteit Amsterdam, 1081 HV Amsterdam, The Netherlands; 5 CIC bioGUNE, Basque Research and Technology Alliance (BRTA), Bizkaia Technology Park, 801 Bld, 48160 Derio, Bizkaia Spain; 6 Ikerbasque, Basque Foundation for Science, 48011 Bilbao, Spain; 7 Centro de Investigación Biomédica en Red de Cáncer (CIBERONC), Madrid, Spain; 8 Université de Lorraine, UAR2008 IBSLor CNRS-UL-INSERM, Biopôle UL, 9, Avenue de La Forêt de Haye, 54505 Vandoeuvre-Les-Nancy, France; 9 Bioinformatics Unit, Cancer Research Center (CIC-IBMCC, CSIC/USAL), Consejo Superior de Investigaciones Científicas (CSIC) and University of Salamanca (USAL), 37007 Salamanca, Spain; 10 Josep Carreras Leukaemia Research Institute (IJC), Badalona, 08916 Barcelona, Catalonia Spain; 11 Germans Trias I Pujol Health Science Research Institute, Badalona, 08916 Barcelona, Catalonia Spain; 12 Department of Pathology, Basurto University Hospital, 48013 Bilbao, Spain; 13 Department of Urology, Basurto University Hospital, 48013 Bilbao, Spain; 14 Traslational Prostate Cancer Research Lab, CIC bioGUNE-Basurto, Biocruces Bizkaia Health Research Institute, Avenida Montevideo 18, 48013 Bilbao, Spain; 15 Carlos III Networked Proteomics Platform (ProteoRed-ISCIII), Madrid, Spain; 16 Centro de Investigación Biomédica en Red de Enfermedades Hepáticas y Digestivas (CIBERehd), Madrid, Spain; 17 Bioinformatics Unit, Spanish National Cancer Research Centre (CNIO), 28029 Madrid, Spain; 18 Servicio de Transgénesis, Nucleus, Universidad de Salamanca, 37007 Salamanca, Spain; 19 Université de Lorraine, UMR7365 IMoPA CNRS-UL, Biopôle UL, 9, Avenue de La Forêt de Haye, 54505 Vandoeuvre-Les-Nancy, France; 20 Institute of Pharmaceutical and Biomedical Sciences, Johannes Gutenberg-University Mainz, Mainz, Germany; 21 Molecular Biotechnology Center (MBC), Department of Molecular Biotechnology and Health Sciences, University of Turin, 10126 Turin, TO Italy; 22 William N. Pennington Cancer Center, Renown Health, Nevada System of Higher Education, Reno, NV 89502 USA; 23 Biochemistry and Molecular Biology Department, University of the Basque Country (UPV/EHU), P. O. Box 644, 48080 Bilbao, Spain

**Keywords:** Epitranscriptome, RNA modifications, Prostate cancer, 7-methylguanosine, tRNA fragments, Tumour microenvironment (TME), Interferon, Immune checkpoint blockade

## Abstract

Newly growing evidence highlights the essential role that epitranscriptomic marks play in the development of many cancers; however, little is known about the role and implications of altered epitranscriptome deposition in prostate cancer. Here, we show that the transfer RNA N^7^-methylguanosine (m^7^G) transferase METTL1 is highly expressed in primary and advanced prostate tumours. Mechanistically, we find that *METTL1* depletion causes the loss of m^7^G tRNA methylation and promotes the biogenesis of a novel class of small non-coding RNAs derived from 5'tRNA fragments. 5'tRNA-derived small RNAs steer translation control to favour the synthesis of key regulators of tumour growth suppression, interferon pathway, and immune effectors. Knockdown of *Mettl1* in prostate cancer preclinical models increases intratumoural infiltration of pro-inflammatory immune cells and enhances responses to immunotherapy. Collectively, our findings reveal a therapeutically actionable role of METTL1-directed m^7^G tRNA methylation in cancer cell translation control and tumour biology.

**Supplementary Information:**

The online version contains supplementary material available at 10.1186/s12943-023-01809-8.

## Introduction

Prostate cancer (PCa) is the second most frequently diagnosed cancer worldwide and the second leading cause of cancer-related deaths in men [1]. Conventional therapies target the hormonal axis of the disease; however, up to 30% of patients eventually develop resistance to therapy and metastasis [2], for which limited treatment options exist. With the recent advent of massive parallel sequencing of tumour samples, molecular profiling efforts have revealed a highly diverse genomic, epigenomic, and transcriptomic landscape [3–7], highlighting the need to identify alternative altered and targetable molecular pathways with therapeutic potential.

Growing evidence implicates a key role for non-mutational mechanisms, such as epigenetics and epitranscriptomics, in the survival and growth of cancer cells [8–11]. Over 170 chemical modifications of RNA are known to compose “the epitranscriptome” [12]. However, their function remains still widely unknown. In recent years, a growing body of evidence has revealed that aberrant deposition of RNA modifications contributes to cancer progression [11, 13, 14].

RNA modifications are prevalent in transfer RNA (tRNAs). Currently, growing evidence has shown that dysregulation of tRNA and its modifying enzymes is also involved in oncogenesis [15–24]. N^7^-methylguanosine (m^7^G) is one of the most prevalent tRNA modifications in eukaryotes and is found in the variable loop region of several tRNA species [25–27]. In humans, m^7^G is catalysed by the complex formed by Methyltransferase1 (METTL1) and the regulatory unit WD Repeat Domain 4 (WDR4) [23, 27–31]. Functionally, m^7^G-modified tRNAs selectively modulate the translation of specific transcripts [23, 27–29]. At the pathological level, increased *METTL1* expression is associated with tumour aggressiveness in several cancer types [22, 23, 28, 29, 32–40]. These observations indicate the critical function of tRNA modifications in cancer development, and suggest that targeting aberrant post-transcriptional modifications in cancer may hold promise as an efficient therapeutic target. Recent developments in RNA methyltransferase inhibitors have shown promising results in preclinical models [11, 16, 24, 41].

Here, we aimed to identify the RNA modifications associated with PCa progression. Our analysis revealed that overexpression of the RNA methyltransferase METTL1 was linked to PCa. We show that loss of guanosine-7 tRNA methylation is crucial in facilitating the biogenesis of a new class of tRNA-derived small RNAs that favour protein synthesis of a distinctive set of genes, and control cancer cell growth and immune responses. We found that decreased *METTL1* expression in human and mouse PCa indicates a better immunotherapy response. Our study uncovers the complex role of the tRNA m^7^G methylome in PCa and highlights the potential of targeting METTL1 as a novel therapeutic strategy to treat PCa.

## Results

### *METTL1* is elevated in human and murine PCa

**Fig. 1:**
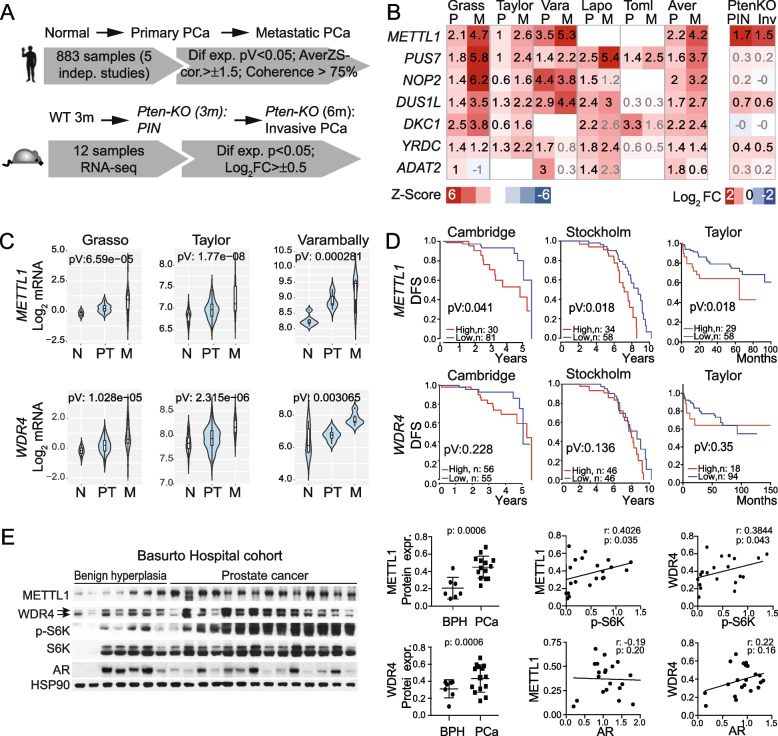
*METTL1* is upregulated in prostate cancer. **A** A schematic overview of the research workflow used to identify altered expression of RMPs associated with PCa. **B** Heatmap of average Z-scores of mRNA expression values in human primary (P) and metastatic (M) PCa samples compared to healthy tissue for significant differentially expressed RNA-modifying enzymes reveals *METTL1* as the most overexpressed RMPs in PCa. Data from are from Grasso et al*.* [7] (*n* = N: 12, P: 49, M: 27); Taylor et al. [6] (*n* = N: 29, P: 131, M: 19); Varambally et al*.* [42] (*n* = N: 6, P: 7, M: 6); Lapointe et al*.* [44] (*n* = N: 9, P: 13, M: 4); Tomlins et al. [43] (*n* = N: 23, P: 32, M: 20) (left panel) datasets. Z-score averages for all datasets are also shown as “Aver”. Z-score values are indicated using numeric values. Grey Z-score values indicate no significant *p*-value. The right heatmap shows the average log_2_ fold change in mRNA expression values in *Pten-cKO* mice with prostate intraepithelial neoplasia (PIN) and invasive prostate carcinoma (Inv) compared to normal prostate tissue (right panel) (*n* = 4). **C** *METTL1* and *WDR4* expression are increased in primary (PT) and metastatic tumours (M) compared to normal tissues (N). Data are from Grasso et al. [7], Taylor et al*.* [6], and Varambally et al*.* [42] datasets. Log_2_-normalised gene expression values are shown. **D** High expression of *METTL1* but not *WDR4* is associated with poor patient prognosis. Kaplan–Meier curves representing biochemical recurrence-free survival (DFS) of patient groups selected according to gene expression, data from the Cambridge, Stockholm and Taylor cohorts [6, 46]. Data were retrieved from the camcAPP [48] and cBioportal. **E** Western blot from benign prostatic hyperplasia (BPH) (*n* = 7) and PCa patient samples (*n* = 14) (upper panel) and correlation analysis between METTL1 and WDR4 expression, AR and phospho-S6K (right panels). Statistical tests: ANOVA test (**B, C**), and log-rank Cox test (**D**). Data are represented as mean ± standard deviation (SD). Student’s *t*-test and Spearman’s correlation test (**E**)

To study the potential role of RNA modifications in PCa tumorigenesis, we followed an approach that ensured the selection of relevant RNA modifications associated with PCa tumorigenesis. We focused on identifying alterations in the expression of a set of 132 annotated RNA-modifying proteins (RMPs) (Supplementary Table S1) in datasets from five PCa studies [6, 7, 42–44] (Fig. 1A). In addition, we extended our analyses to murine PCa using a genetically engineered PCa mouse model [45] (Fig. 1A). We found that the most strikingly differentially expressed gene in primary and metastatic PCa was *METTL1* (Fig. 1B; Supplementary Fig. S1A, Supplementary Table S1). *METTL1* expression consistently increased from primary to metastatic human tumours (Fig. 1C), and high expression of *METTL1* showed worse prognosis in Cambridge, Stockholm, and Taylor cohorts [6, 46] (Fig. 1D; Supplementary Fig. S1B). We also found elevated expression of *WDR4* (Fig. 1C)*,* the regulatory subunit of the m^7^G RNA methyltransferase complex [30, 31], which is also overexpressed in other cancers [22, 28, 29, 38, 39]; however, we did not find *WDR4* overexpression to be a risk factor for poor prognosis (Fig. 1D; Supplementary Fig. S1C). Increased expression of *METTL1* was confirmed in PCa-derived cell lines compared to immortalised benign prostatic hyperplasia or prostatic epithelium-derived cell lines (Supplementary Fig. S1D, S1E). We did not find the same correlation of higher expression of *WDR4* in PCa cell lines (Supplementary Fig. S1D, S1E). Increased protein expression of METTL1 and WDR4 was confirmed in PCa samples from a local cohort (Basurto Hospital) (Fig. 1E). Based on the hormonal dependency of PCa tumours, expression analysis of METTL1, WDR4 and AR was performed, but no significative correlation was found between METTL1, WDR4 and AR expression (Fig. 1E). To confirm whether METTL1 and WDR4 expression could correlate with advanced tumour status, we measured PI3K pathway activity by phosphorylation status of S6K, which altered in in approximately 70% of patients with advanced PCa [47]. We found a positive and significant correlation between METTL1 and WDR4 expression and PI3K enhanced pathway activation (Fig. 1E), indicating that METTL1 and WDR4 expression is increased in advanced PCa tumours.

In summary, our results demonstrate that METTL1 is the main epitranscriptomic regulator altered in PCa, and its overexpression is correlated with poor prognosis.

### *METTL1* expression is regulated by AKT-mTOR downstream signalling pathway

**Fig. 2:**
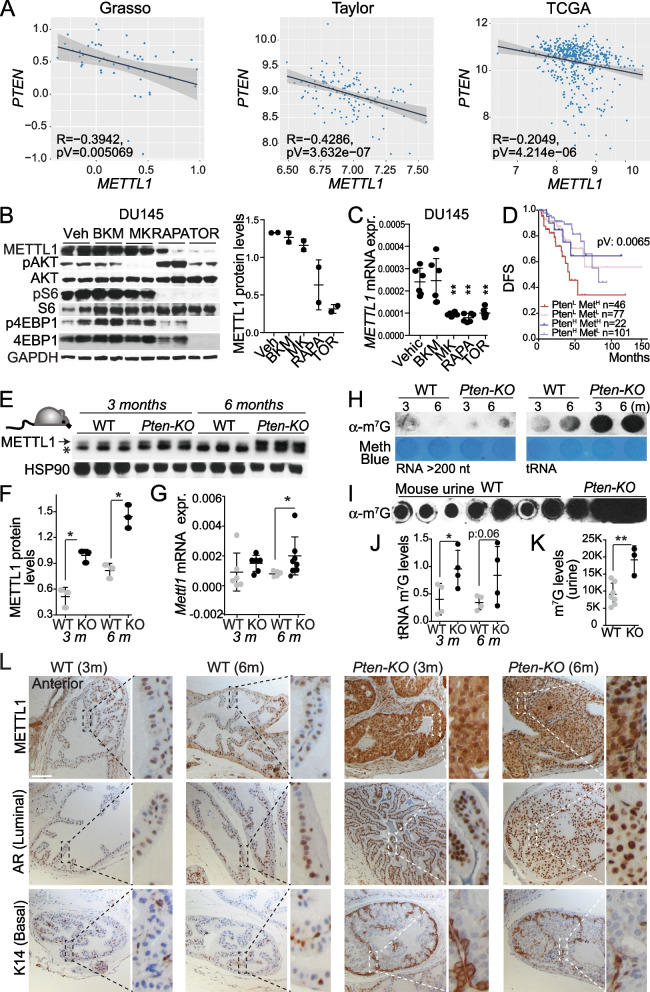
PI3K-AKT-mTORC pathway mediate upregulation of *METTL1* expression in PCa. **A** Correlation analysis between *METTL1* and *PTEN* expression in human primary prostate tumours expression datasets. Plotted values correspond to the log_2_-normalised gene expression values for each patient in the indicated dataset. The black line represents linear regression, grey area indicates the limits of the confidence intervals. Pearson’s correlation coefficient (R) and *p*-values are indicated. Grasso *n* = 88; Taylor *n* = 183; TCGA *n* = 497. **B, C** *METTL1* expression is regulated downstream of AKT signalling. Western blot (**B**) and RT-qPCR (**C**) analyses of *METTL1* expression upon PI3K pathway inhibition in DU145 cells. DMSO as vehicle (Veh), BKM-120 (BKM) as pan-PI3K inhibitor, MK2206 (MK) as AKT inhibitor, rapamycin (RAPA) as mTORC1 inhibitor, and Torin (TOR) as mTORC1/2 inhibitor. For western blotting, cells were treated for 48 h, and for RT-qPCR, for 8 h. Means ± SD are shown (*n* = 2) (**B**) and (*n* = 6) (**C**). **D** Stratification of patients with a worse prognosis according to high *METTL1* levels and low *PTEN* expression. Kaplan–Meier curves representing the disease-free survival (DFS) of patient groups selected according to Q1 (Pten^L^) and Q4 (Pten^H^) quartile expression of *PTEN*, and *METTL1* high (Met^H^, log_2_-normalised expression > 8.72) and *METTL1* low (Met^L^, log_2_-normalised expression < 8.72) in recurrent and disease-free (DF) tumour samples from the TCGA dataset. **E–G** *Mettl1* is highly expressed in mouse prostate tumours. *Mettl1* expression analysis in *Pten-KO* mice at 3 and 6 months of age compared to wild-type mice (WT) at the same ages by western blot (**E, F**) and RT-qPCR (**G**). * in (**E**) indicates an unspecific band. Means ± SD are shown for three (**F**) and five replicates (**G**). **H, J** Increased 7-guanine tRNA methylation in RNA from prostate tumours compared with that in normal prostate. North-dot blot of m^7^G levels in long RNAs (> 200 nucleotides long RNAs) and tRNAs extracted from prostatic tissue of *Pten-KO* mice with intraepithelial prostatic neoplasia (at 3 months of age) or invasive tumour (at 6 months), and wild-type (WT) mice of the same age. Methylene blue staining was used as the loading control (**H**, bottom panel). Means ± SD are represented (*n* = 4) (**J**). **I, K** Increased 7-guanine tRNA methylation in the urine of mice bearing PCa tumours. North-dot blot of m.^7^G levels from the urines of *Pten-KO* mice with invasive tumour (at 6 months), and wild-type (WT) mice of the same age. Means ± SD are shown (n > 3). **L** Representative images of immunostained sections for Mettl1 and markers for luminal (AR), and basal (K14) cells in *Pten-KO* prostates (anterior lobes) at initiation (3 m) and in invasive carcinoma (6 m) and in age-matched wild-type (WT) prostates*.* Scale bars represent 50 μm. Statistical tests: one-tailed Student’s t-test (**C**), log-rank test (**D**), Mann–Whitney test (**F, G, J, K**). *p < 0.05, **p < 0.01, ***p < 0.001

Next, we sought to elucidate the mechanism underlying the upregulation of *METTL1* expression in PCa. Because increased androgen receptor (AR) activity is one of the main PCa drivers, we analysed whether increased AR expression or activity, measured by the expression of the receptor and downstream target genes such as *KLK3*, correlated with *METTL1* expression in PCa. In the Grasso dataset [7], we observed an almost significant direct correlation between *METTL1* and both *AR* and *KLK3*. In the Taylor dataset [6], we found a direct correlation between *METTL1* and *AR*, supporting the notion of a potential relationship between these two factors. However, in the case of *KLK3*, we did not observe a significant correlation. Our analysis using the TGCA dataset revealed a direct correlation between *METTL1* and *KLK3* [3]. However, interestingly, we did not find a significant correlation between *METTL1* and *AR* in this particular dataset. This inconsistency suggests that the association between *METTL1* and *KLK3* may vary depending on the dataset being analysed, indicating a lack of robustness or consistency in the observed correlation (Supplementary Fig. S2A). To further clarify whether METTL1 expression was regulated under the AR pathway, we measured the mRNA levels of *METTL1* after dihydrotestosterone (DHT) treatment in the androgen-sensitive PCa cell line, LNCaP, and found no changes (Supplementary Fig. S2B), suggesting that *METTL1* expression is not regulated by AR activity. Because we found a positive correlation between METTL1 protein expression and phospho-S6K in PCa samples compared to benign prostatic hyperplasia specimens (Fig. 1E), we next analysed whether *METTL1* expression was correlated with *PTEN* expression, a negative regulator of the PI3K/AKT/mTOR pathway, which is deleted in approximately 70% of patients with advanced PCa [47]. All datasets analysed showed a significant negative correlation between *METTL1* and *PTEN* expression (Fig. 2A), suggesting that the PI3K-mTOR axis regulates *METTL1* expression in PCa. Further dissection of the PI3K–mTORC1/2 pathway using small-molecule inhibitors of PI3K (BKM-120 inhibitor), AKT (MK2206), mTORC1 (rapamycin), and mTORC1/2 (Torin) revealed that AKT inhibition decreased *METTL1* mRNA levels, and mTOR inhibitors consistently decreased *METTL1* mRNA and protein expression (Fig. 2B-C; Supplementary Fig. S2C-E), indicating that *METTL1* expression is regulated via mTOR signalling.

Next, we examined whether *METTL1* expression levels helped to define patients with *PTEN*-loss-associated decreased survival, which is of clinical relevance, and distinguish indolent from aggressive disease in patients with clinically localised tumours [47]. Interestingly, we found that high *METTL1* expression highlighted a subset of PTEN-low patients with poor outcomes (Fig. 2D).

To ascertain whether *METTL1* upregulation in PCa was a direct consequence of low or loss of *PTEN* expression, we analysed *Mettl1* mRNA and protein levels in prostate tissues from wild-type (WT) mice and *Probasine-Cre* x *Pten*^*flox/flox*^ mice (hereafter *Pten-KO),* with conditionally have deleted *Pten* in the prostate epithelium. These mice develop high-grade pre-neoplastic lesions after 12 weeks, which progress to invasive adenocarcinoma after 5 months of age [45]. We observed a progressive increase in *Mettl1* expression after *Pten* deletion (Fig. 2E-G). Interestingly, tRNAs from mouse prostate tumours showed significantly increased deposition of m^7^G in tRNA extracted from tissues, as well as in urine (Fig. 2H-K). Immunohistochemical analysis further revealed that *Mettl1* expression was higher in murine PCa and was highly expressed in luminal cells, which are the most common epithelial cell type and are generally accepted as the preferred cell of origin for human prostate cancer [49] (Fig. 2L; Supplementary Fig. S2F, S2G).

Taken together, these data indicate that METTL1 is a downstream effector of the mTORC1 pathway and its activation induces increased expression of METTL1 in PCa.

### METTL1 preferentially methylates tRNAs

**Fig. 3:**
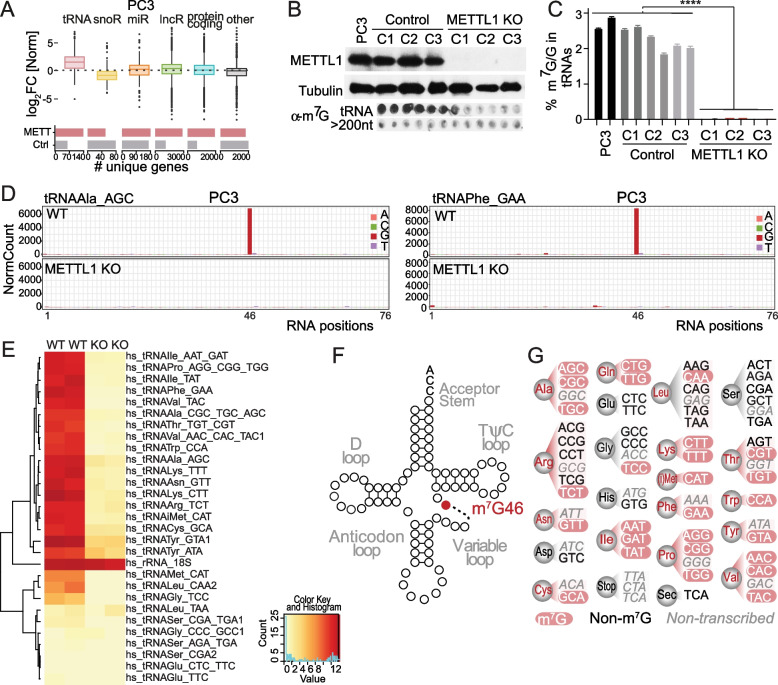
METTL1 preferentially methylates tRNAs. **A** tRNAs are the most common RNA species bound to METTL1. Upper panel: Boxplot representing the median Log_2_ fold change of reads per million (RPM) per transcript bound to METTL1 in PC3 cells. Lower panel: The bar plot shows the total number of unique genes bound to METTL1. **B** Validation of the lack of METTL1 expression and m^7^G tRNA methylation in PC3 *METTL1 KO* cell lines. Western blot of METTL1 (upper panel) and north-dot blot of m^7^G levels (lower panel). Proteins, long RNAs (> 200 nucleotides), and tRNAs (< 200 nucleotides) were extracted from three independent PC3 *METTL1 KO* clones and three control clones. Parental PC3 cells are shown. **C** LC–MS analysis of m^7^G levels in tRNAs isolated from PC3 *METTL1 KO*, control, and parental PC3 cells validate the absence of m^7^G in tRNAs extracted from METTL1 KO cells. Means ± SD are represented (*n* = 3). **D** Normalised cleavage signals for the tRNAs AlaAGC and PheGAA in PC3 WT and *METTL1 KO* cells. Letters on the right show the bases where methylation occurs. **E** Heatmap showing normalised cleavage values for all tRNAs with guanosines at position 46 in PC3 WT and *METTL1 KO* cells (*n* = 2 for each genotype). **F** tRNA secondary structure showing METTL1-methylated guanines (red circles) in the variable loop. **G** Graphical summary of tRNA isoacceptors methylated by METTL1 (red) and non-methylated or non-transcribed (grey). Statistical tests: two-tailed Student’s t-test (**C**). ****p < 0.0001

To understand the role of METTL1 in PCa tumorigenesis, we determined METTL1 RNA substrates by combining two transcriptome-wide methodologies. To identify METTL1-specific RNA targets in PCa cells, we used photoactivatable-ribonucleoside-enhanced cross-linking immunoprecipitation (PAR-CLIP), a stringent technique that incorporates photoreactive ribonucleoside analogues into nascent RNAs that are UV-crosslinked to induce a covalent bond between proteins and RNA, followed by next-generation sequencing [50]. We generated PC3 cells expressing doxycycline-inducible HA-METTL1 and used them to immunoprecipitate HA-METTL1 (Supplementary Fig. S3A). Doxycycline-induced cells infected with the empty vector were used as control. We found that tRNAs represented the most enriched RNA species bound to METTL1 when compared to those RNAs bound to the control sample, and with the highest density of reads per gene, with an average median density of Log_2_ 12 RPMs. (Fig. 3A; Supplementary Fig. S3B; Supplementary Table S2). We did not observed an enrichment of other RNA species bound to HA-METTL1 samples and compared to control samples (Fig. 3A; Supplementary Fig. S3C). To further validate these results, we downloaded the data generated by *Bao *et al. [51] and analysed the METTL1-bound RNAs in HEK293T cells. tRNAs were also the most abundant RNA species bound to METTL1 in HEK293T cells, with an average median density of Log_2_ 8 RPMs. The total number of reads mapped to other noncoding RNAs (ncRNAs) and mRNAs was respectively 20% for ncRNAs and approximately 16% for mRNAs, with a median density lower than 16 RPMs per gene, which we considered background (Supplementary Fig. S3D-F; Supplementary Table S3).

Next, to precisely map m^7^G in tRNAs, we knocked out *METTL1* in the PCa cell line PC3 using CRISPR/Cas9 (Fig. 3B; Supplementary Fig. S3G, S3H). North-dot blot analysis using anti-m^7^G antibodies and mass spectrometry analysis confirmed the loss of m^7^G in tRNAs from *METTL1 KO* cells (Fig. 3B-C). We then performed AlkAnaline-seq to map m^7^G occurrence at single-nucleotide resolution and used *METTL1 KO* tRNAs as controls (Supplementary Fig. S3I) [52]. Analysis of high-throughput sequencing data corroborated the robust methylation in the variable loop at guanosine 46 as previously reported [23, 26–28, 53] (Fig. 3D, F; Supplementary Fig. S3J). We identified approximately 50% of all isoacceptors as METTL1 substrates (Fig. 3E, G). Taken together, our analyses corroborated that METTL1 preferentially methylates tRNAs at the variable loop in PCa cells.

### METTL1-mediated methylation protects tRNAs from cleavage into small noncoding RNAs

**Fig. 4:**
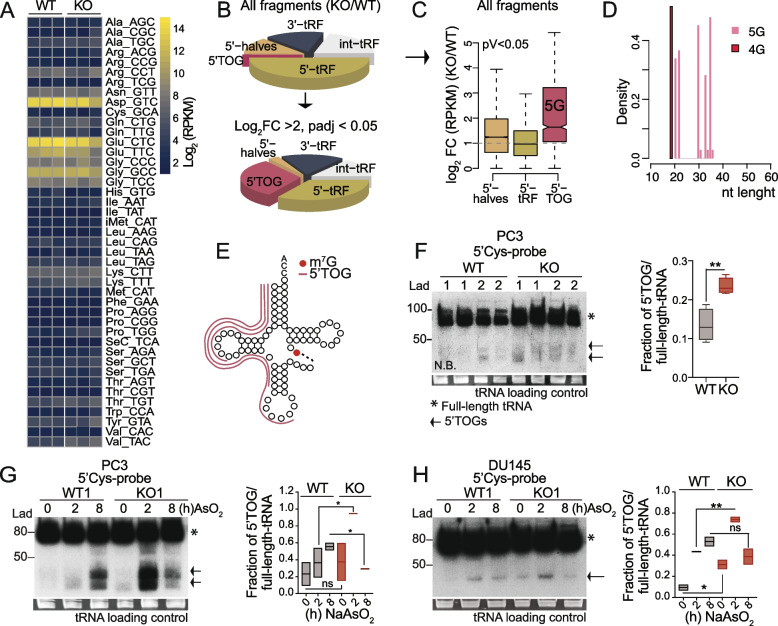
The lack of m^7^G methylation in tRNAs leads to 5'tRNA fragment accumulation. **A** Heatmap showing no differences in the levels of mature tRNAs (log_2_ normalised reads; RPKM) measured using tRNA-seq data. Rows represent individual tRNA isoacceptors and columns represent independent replicates. tRNA isoacceptor expression levels are row-scaled (each row is normalised to their mean expression and standard variation). **B** The top pie chart represents the percentage of all abundant tRNA fragments identified in PC3 *METTL1 KO* cells compared to that in WT cells. The bottom pie chart represents the percentage of differentially express tRNA fragments: fragments with log_2_ fold change (FC) > 2 and *p* value < 0.05 in PC3 *METTL1 KO* cells versus WT cells. The pie charts show that while 5'tRNA fragments are very abundant in KO cells, 5'TOGs are significantly overexpressed in KO cells compared to WT cells. 3'tRFS: 3' tRNA fragments; Int-tRFs: internal tRFs; 5'tRNA: 5'tRNA fragments > 18 and < 35 nucleotides; 5'-halves: 5'tRNA fragments > 35 nucleotides; 5'TOGs: 5'tRNA fragments > 18 and < 35 nucleotides, with 5′ terminal oligoguanine. **C** The boxplot shows the log_2_ fold change (FC) of all fragments (not just the differentially expressed tRNA fragments) in PC3 *METTL1 KO* versus WT cells. **D** Size and abundance (density) of 5'TOGs Cys-derived (5G) or Ala-derived (4G) fragments differentially expressed (log_2_ FC > 1.5, > 18 nt, and *p* < 0.05) in PC3 *METTL1 KO* cells versus WT cells. **E** Summary of 5'TOGs formed in PC3 *METTL1 KO* cells. **F** Increased tRNA fragmentation was observed by northern blot detection of Cys-derived 5’TOGs in PC3 *METTL1 KO* vs. WT cells (2 technical replicates of 2 biological replicates are represented) (left panel). The boxplot of the right shows quantification of densities of Cys-derived 5'TOGs formed versus full-length tRNAs. **G, H** Cys-derived 5'TOG fragments are induced by stress. Northern blot detection of Cys-derived 5'TOG in PC3 and DU145 *METTL1 KO* and WT cells unexposed (0 h), or after 2 and 8 h of oxidative stress exposure. The boxplots of the right show the fraction of Cys-derived 5'tRFs formed normalised to the fraction of full length tRNAs. Values are from two biological replicates, shown in this figure and in Supplementary Fig. S4G and S4J. The loading control of tRNA is shown in the bottom panel as red-safe staining (**F–H**). Bands corresponding to full length tRNAs are indicated with stars and 5'tRNA fragments are indicated with arrows. Statistical tests: one-tailed Student’s t-test. **p < 0.01 (**F–H**)

The stability of tRNAs decreases when m^7^G methylation is reduced in yeasts and humans [22, 23, 27, 28, 54]. To establish that *METTL1* loss might perturb the levels of individual METTL1-targeted tRNAs in PCa cells, we performed high-throughput sequencing of the tRNAs isolated from PC3 WT and *METTL1 KO* cells. In contrast to previous findings, we found no evidence that the loss of METTL1-specific methylation reduces the abundance of specific mature tRNA isoacceptors (Fig. 4A).

Recent reports indicate that tRNA modifications protect or induce cleavage of tRNAs into inhibitory small ncRNAs [55–59]. We analysed the small RNA sequencing data from PC3 WT and *METTL1 KO* cells for differences in the major categories of tRNA fragments (tRFs), including 5′/3′-derived tRFs (5′tRF and 3′tRF), 5′tRNA halves, and internal tRFs (int-tRF) [58]. Interestingly, we detected consistent enrichment of 5′tRNA fragments in PC3 *METTL1 KO* cells (Fig. 4B; top panel; Supplementary Table S4); however, most of them showed a modest increase in enrichment in *METTL1* KO cells compared to WT cells (Fig. 4C). Our analysis revealed that from all 5′tRNA fragments, a specific class, known as 5′terminal oligoguanine-containing tRNA fragments (5′TOGs), was significantly overrepresented (log_2_ FC > 2, *p* value < 0.05) in KO cells compared to WT cells (Fig. 4B, bottom panel; Fig. 4C) [57, 60]. The 5′TOGs were ~ 20 or ~ 30 nucleotides long and were mainly derived from the cleavage of the METTL1-target tRNA Cys (which gave rise to 5 terminal guanines (5G) containing 5′TOGs) and Ala (which gave rise to 4G containing 5′TOGs) (Fig. 4D-E; Supplementary Fig. S4A). Northern blotting confirmed a weak but significantly higher accumulation of Cys-derived 5′tRFs in two independent clones of PC3 *METTL1 KO* cells compared to WT cells (Fig. 4F). 5′tRFs were observed upon *METTL1* downregulation in PC3, DU145 and 22Rv1 cells, indicating that 5′tRFs formation is independent of PTEN, p53 and AR status (Fig. 4F; Supplementary Fig. S4D-F, S4I, S4J). We did not detect the accumulation of 5′tRNA fragments derived from other METTL1-tRNA targets, such as Lys or Pro tRNAs (Supplementary Fig. S4B, S4C; Supplementary Table S4), suggesting that only a subset of METTL1 tRNA targets are cleaved when hypomethylated.

Compelling evidence has revealed that the cleavage of a small fraction of tRNAs into tRNA-derived ncRNAs is a conserved response to stress, and tRNA modifications protect them from stress-induced cleavage [55, 58, 61–65]. During stress responses, tRNA cleavage was more prominent in *METTL1 KO* cells than in WT cells, peaking as early as two hours of oxidative stress exposure, and decreasing after 8 h of stress stimulus, probably because of an increase in cell death due to inability to resolve the stress (Fig. 4G, H; Supplementary Fig. S4G, S4J). We did not detect the accumulation of 5′tRNA fragments derived from other METTL1 tRNA targets (i.e. Lys) upon oxidative stress induction (Supplementary Fig. S4H), indicating that 5′tRF formation after stress is tRNA-specific.

In summary, our data indicate that METTL1-mediated methylation is a conserved mechanism that regulates the biogenesis of a novel class of small ncRNAs derived from 5′tRNA fragments during stress responses in PCa cells, regardless their genetic status.

### Loss of *METTL1* represses translation initiation through tRNA fragment biogenesis

**Fig. 5:**
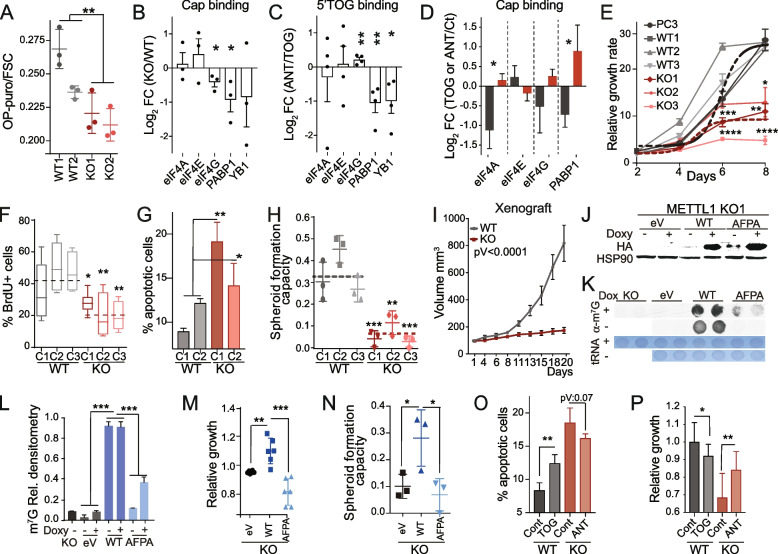
*METTL1* downregulation suppresses protein synthesis, proliferation and tumour growth in vivo. **A** Global protein synthesis rate measured by flow cytometry analysis of OP-puromycin (OP-puro) incorporation reflects reduced protein synthesis in PC3 *METTL1 KO* cells compared to the control (WT). Fluorescence was normalised to cell size (FSC) in WT and *METTL1 KO* cells. Two biological and three technical replicates and the mean ± SD are shown. **B** Translation initiation and regulatory factors are displaced from the cap of mRNAs in *METTL1* KO cells. Log_2_ fold change (FC) binding of the indicated translation initiation and regulatory factors to m^7^G-cap-coated sepharose beads in PC3 *METTL1 KO* vs. WT cells. Densitometry data were normalised to the input. Mean ± SEM, *n* = 3. **C** Anti-TOG RNAs block the 5'TOG-dependent displacement of translation initiation factors in vitro. Log_2_ FC of translation initiation factors bound to synthetic biotinylated-5'TOG in PC3 WT cells transfected with 5'TOG + Anti-TOGs (ANT) compared to PC3 WT cells transfected with 5'TOG + scramble RNAs (TOG). Densitometry data were normalised to the input. Mean ± SEM, *n* = 4. **D** Displacement of translation initiation factors from mRNA caps is TOG-dependent and can be reversed by expressing anti-TOG RNAs. Log_2_ fold change (FC) of m^7^G-cap-bound translation initiation factors in PC3 WT and *METTL1 KO* cells transfected with biotinylated-5'TOG (TOG) or anti-TOG RNA (ANT) versus cells transfected with scramble RNA oligonucleotides. Densitometry data were normalised to the input. Mean ± SEM, *n* = 3. Original wester blots are shown in supplementary figure S5 (**B, C, D**). **E** Growth curves of PC3 *METTL1 KO*, WT, and parental cells (PC3). Mean ± SD, *n* = 3. The dotted line represents the average growth of WT and parental or KO cells. **F** Reduced cell division rate, as measured by BrdU incorporation. **G** *METTL1* depletion increased apoptosis in PC3 *METTL1 KO* cells. Flow cytometry analysis of Annexin V staining. Mean ± SD, *n* = 3. **H** Reduced spheroid formation capacity in PC3 *METTL1 KO* cells. Means ± SD, *n* = 3. The dotted line represents the average values of all WT or KO cells. **I** Tumour growth in xenografted PC3 *METTL1 KO* and WT cells in athymic nude mice reflects impaired tumour formation in the absence of *METTL1*. Mean ± SEM, *n* = 10. **J-L** Protein expression (**J**) and m^7^G methylation levels of tRNAs (**K, L**) of PC3 *METTL1 KO* cells ectopically expressing a doxycycline-inducible HA-tagged wild-type (WT) or a catalytic dead mutant (AFPA) version of METTL1. PC3 *METTL1 KO* cells were infected with an empty vector (eV) as a control. Methylene blue staining was used as the loading control (**L**, bottom panel). Mean ± SD, *n* = 3 (**L**). **M, N** Proliferation (**M**) and spheroid formation capacity (**N**) were dependent on METTL1 catalytic activity. PC3 *METTL1 KO* cells re-expressing METTL1 (WT) or catalytic dead mutant (AFPA) compared to *METTL1 KO* cells infected with empty vector (eV). Mean ± SD, *n* = 6. **O, P** 5'TOG transfection induces apoptosis and reduces cell proliferation. Percentage of apoptotic (**O**) and growth rates (**P**) of PC3 *METTL1 KO* and WT cells after transfection with synthetic 5'TOGs (TOG) or anti-TOG (ANT) RNAs. Controls (Cont) were transfected with scramble RNAs. Mean ± SD, *n* = 6 (**O**), n ≥ 10 (**P**). Statistical tests: Two-way ANOVA (**E, I, L**), one-way ANOVA (**F, G, H**), and one-tailed Student’s t-test (**A-D, M-P**). *p < 0.05, **p < 0.01, ***p < 0.001, ****p < 0.0001

As 5′TOGs are known to repress global translation [57, 60, 62], we next investigated whether the accumulation of 5′TOGs contributed to translation alterations in PC3 *METTL1*-depleted cells. We confirmed a reduction in protein translation measured by O-propargyl-puromycin (OP-puro) incorporation in *METTL1 KO* cells compared to that in WT cells (Fig. 5A). The changes in protein synthesis rates were not associated with expression changes of components of the translation initiation complex or in the phosphorylation status of eIF2α (Supplementary Fig. S5A).

The production of 5'tRNA fragments in response to cellular stress play a critical role in the stress response mechanism. This process helps to inhibit global protein synthesis, allowing the cells to recover and survive stressful conditions [66]. To determine whether the removal of *METTL1* impacts the stress response, we measured protein synthesis rates after oxidative stress induction. Our analysis showed that *METTL1*-deficient cells displayed a reduced recovery rate of protein synthesis when compared to WT cells, indicating that *METTL1* KO cells are more sensitive to stress (Supplementary Fig. S5B). This finding suggests that the loss of m^7^G significantly repressed protein synthesis and this process is independent of eIF2α phosphorylation status.

Next, we sought to define the molecular basis for the distinctive translational repression of *METTL1 KO* cells and the dependence on 5′TOGs. Previous evidence has shown that 5′TOGs impair translation initiation by displacing the components of the translation initiation complex eIF4A/G/E and regulatory factors YB1 and PABP1 from the cap of mRNAs [57, 60, 67]. To determine whether translation initiation factors were displaced from 7-methyl-guanylated (m^7^G)-capped mRNAs in PC3 *METTL1 KO* cells, we analysed the affinity of translation initiation factors for m^7^G-cap-coated sepharose beads. We found that the m^7^G-caps affinity of eIF4G and PABP1 was significantly decreased in *METTL1 KO* cells compared to that in WT cells (Fig. 5B; Supplementary Fig. S5C). This suggested that the increased accumulation of 5′TOGs destabilised the assembly and binding to the cap of some factors of the translation initiation complex, leading to repressed translation in *METTL1 KO* cells.

We further evaluated whether synthetic 5′TOGs expressed in WT cells were able to bind to translation initiation and regulator factors and whether their affinity to these factors could be blocked using synthetic reverse-complement 5′TOG RNAs (or anti-TOGs). To this end, we transfected PC3 WT cells with 5′biotinylated-5′TOG RNAs (containing the sequence of the most abundant 5′TOG found in PC3 *METTL1 KO* cells) with or without the addition of anti-TOGs. After pulldown of 5′biotinylated-5′TOGs, we found that the affinity of PABP1 and YB1 for 5′TOG was significantly reduced in the presence of anti-TOGs (Fig. 5C; Supplementary Fig. S5D). Next, we investigated whether synthetic 5′TOGs could phenocopy the translation initiation complex displacement in WT cells and whether anti-TOG RNAs could rescue this observed effect in *METTL1 KO* cells. We found that the binding of PAPB1 to m^7^G-caps was displaced in WT cells transfected with 5′TOGS, but the m^7^G-caps affinity of PAPB1 was significantly increased in *METTL1 KO* cells transfected with anti-TOG RNAs (Fig. 5D; Supplementary Fig. S5E). Altogether indicating that the formation of 5′TOGs repress protein translation by displacing PAPB1. Interestingly, PAPB1 has been previously shown to have a strong affinity for 5′TOGs in other cell types [57].

Thus, our results confirm that 5′TOGs expressed in *METTL1 KO* cells can displace translation regulatory factors from mRNA caps and provide key insights into the molecular basis underlying METTL1-mediated translational repression.

### *METTL1* downregulation suppresses prostate tumour growth in vitro and in vivo

Given that *METTL1 KO* cells synthesise fewer proteins, we hypothesised that METTL1 inhibition could reduce cell and tumour growth. In fact, we observed that knocked down of *METTL1* impaired the growth of PC3, DU145 and 22Rv1 cells (Fig. 5E; Supplementary Fig. S6A, S6B). *METTL1* downregulation also diminished cell division, induced cell cycle arrest, increased apoptosis, impaired spheroid formation capacity, and drastically reduced the growth and proliferation of tumour xenografts (Fig. 5F-I; Supplementary Fig. S6C-F). Thus, our data indicated that *METTL1* downregulation can effectively decrease tumour growth, strongly supporting that METTL1 is crucial in the regulation of PCa progression.

Next, we investigated whether METTL1 methylase activity was sufficient to promote cell growth. We re-expressed a wild-type version of METTL1 (WT) and a catalytically dead mutant (AFPA) in PC3 *METTL1 KO* cells (Fig. 5J-L; Supplementary Fig. S6G-I). The proliferation and spheroid formation capacity of *METTL1 KO* cells re-expressing WT METTL1 were increased compared to *KO* cells transduced with an empty vector (*eV*). In contrast, the expression of the catalytically inactive mutant failed to promote *METTL1 KO* cell growth and spheroid formation capacity (Fig. 5M-N; Supplementary Fig. S6J, S6K). Because METTL1 catalytic activity is supported by the formation of a complex with the regulatory subunit WDR4 [30, 31], and WDR4 is overexpressed in PCa (Fig. 1C, E), we tested the oncogenic potential of WDR4. Inducible silencing of *WDR4* did not reduce cell or tumour growth (Supplementary Fig. S6L, S6M). Together, these results suggest that METTL1 promotes the growth of PCa cells in an enzymatic activity-dependent manner, but is independent of WDR4.

To further determine whether 5′TOGs were sufficient to induce growth arrest and apoptosis, we transfected PC3 WT cells with 5′TOG and *METTL1 KO* cells with anti-TOG and measured apoptosis and proliferation. We detected a significant increase in apoptosis and decrease in proliferation when WT cells were transfected with 5′TOG RNAs, and a decrease in apoptosis, although not significant, and a significant increase in proliferation when *METTL1 KO* cells were transfected with anti-TOGs (Fig. 5O, P).

Altogether, our data suggest that METTL1 inactivation and 5′TOGs are required to induce growth arrest, and the re-expression of the catalytically active version of METTL1 and anti-TOGs can partially rescue the observed defects in *METTL1 KO* cells.

### *METTL1* loss activates IFN signalling pathway

**Fig. 6:**
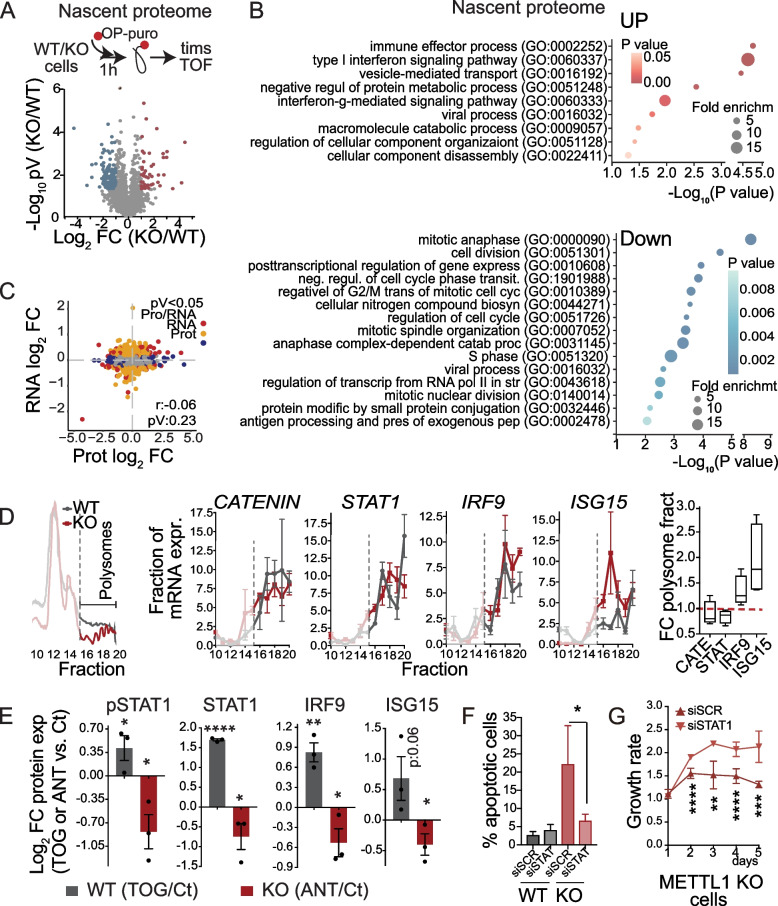
Loss of m^7^G tRNA methylation results in distinct translational programmes. **A** Schematic overview of nascent polypeptide OP-puro-labelling and enrichment followed by LC–MS/MS peptide identification and quantification analysis (upper panel). Lower panel shows common differentially expressed nascent proteins ranked in a volcano plot according to their statistical *p*-value (-Log_10_ pV) and their relative abundance ratio (Log_2_ FC) between four replicates of PC3 WT and *METTL1 KO* cells. Coloured dots represent statistically (*p*-value < 0.05) upregulated (red) and downregulated (blue) proteins in *METTL1 KO* cells. **B** Gene Ontology (GO) category enrichment of biological processes in significantly (*p*-value < 0.05) upregulated (UP, FC > 1) or downregulated (Down, FC < 1) nascent proteins in *METTL1 KO* cells compared to WT cells. Categories were ranked according to their statistical *P*-value (-Log_10_ pV) and fold enrichment of genes found for each category. **C** No correlation between differentially expressed proteins (protein log_2_ FC) and their mRNA (protein log_2_ FC) was observed in PC3 WT vs. *METTL1 KO* cells. Coloured dots represent significant (*p*-value < 0.05) differentially expressed proteins (blue), mRNAs (yellow) or both (red) for each gene. **D** Representative polysome profile in WT (grey line) and *METTL1 KO* PC3 cells (red line) (left panel) shows reduced translation in *METTL1 KO* cells. The fraction of the abundance of each mRNA in each polysome fraction is shown with respect to the content in all fractions, reflecting increased translation of *IRF9* and *ISG15* in *METTL1 KO* cells. Mean ± SEM, *n* = 3. The right boxplot represents the fold change (FC) of mRNA content in the polysome fraction relative to non-polysome fractions. **E** Increased IRF9, ISG15 and STAT1 protein expression observed in PC3 *METTL1 KO* cells is 5'TOG-dependent. Protein expression levels are represented as log_2_ fold change for WT (grey bars) transfected with 5'TOG RNA (TOG) versus scramble control RNA (Ct), and *METTL1 KO* cells (red bars) transfected with anti-TOG RNAs (ANT) versus scramble control RNAs (Ct). Mean ± SEM, *n* = 3. Original western blots are shown in supplementary figure S7. **F, G** STAT1-dependence of apoptosis (**F**) and growth rates (**G**) of *METTL1 KO* and WT PC3 cells. KO cells were transfected with siScramble or siSTAT1. Mean ± SD, *n* = 6 (**F, G**). Statistical tests: One-tailed Student’s t-test was used (**E–G**). *p < 0.05, **p < 0.01, ***p < 0.001

Given that translation is affected by METTL1 inhibition, we explored the consequences of *METTL1* loss on immediate translatome changes. To this end, we took advantage of the OP-puro properties to label nascent proteins that were subsequently conjugated to biotin-coated beads, followed by on-bead digestion and LC–MS/MS (Fig. 6A) [68]. Gene ontology (GO) term enrichment analyses identified decreased translation of genes linked to cell division and mitosis, corroborating the observed defects in proliferation of *METTL1 KO* cells (Fig. 6B; Supplementary Table S5). Unexpectedly, we also found increased translation of transcripts associated with stress responses, including type I and II interferon (IFN) signalling pathways, immune effector processes, and catabolic processes in *METTL1 KO* cells (Fig. 6B). The differences in translation were reflected in the global proteome composition of PC3 *METTL1 KO* cells*,* but did not correlate with RNA expression levels, suggesting that post-transcriptional or translational regulation was responsible for the uncorrelated RNA–protein expression levels found in *METTL1 KO* cells (Fig. 6C; Supplementary Fig. S7A, S7C, S7D; Supplementary Tables S6, S7, S8). To further test whether the translational efficiency of certain transcripts differed in *METTL1 KO* cells, we performed polysome profiling. This approach enabled us to confirm the decreased formation of active polysomes in *METTL1 KO* cells, concomitant with a reduction in global protein synthesis (Fig. 6D). Analysis of mRNA enrichment in the polysome fractions of *METTL1 KO* cells revealed increased translation of specific transcripts linked to interferon signalling, such as interferon regulatory factor 9 (IRF9) and interferon-stimulated gene 15 (ISG15) (Fig. 6D). As a control for specificity in translational changes, we did not find enrichment of transcripts such as *GAPDH,* Catenin β, *KIF20A* or *STAT1* in the polysome fractions of *METTL1 KO* cells (Fig. 6D; Supplementary Fig. S7B). Taken together, our findings indicate that METTL1-mediated tRNA methylation steers a distinct translational programme.

Because 5′TOGs can re-programme the translation machinery to favour the translational programmes required for cancer cells [58, 69], we next investigated whether translational changes in *METTL1 KO* cells were mediated by the increased biogenesis of 5′TOGs. To this end, we transduced PC3 WT and *METTL1 KO* cells with 5′TOG and anti-TOG RNAs and assessed the changes in protein expression. We found that 5′TOGs transfection in WT cells increased IRF9 and ISG15 protein expression, whereas transfection of anti-TOG RNAs in *METTL1 KO* cells slightly but significantly decreased the expression of both proteins (Fig. 6E; Supplementary Fig. S7E). We also observed increased protein expression and phosphorylation of STAT1 in *METTL1 KO* cells and in xenografts (Fig. 6E; Supplementary Fig. S7E, S7F), yet its increased expression was independent of increased translation (Fig. 6D), but was transcriptionally dependent (Supplementary Fig. S7G), suggesting that *STAT1* increased expression was induced by activation of the IFN signalling pathway in *METTL1 KO* cells [70, 71]. In fact, the transcription of other interferon-stimulated genes (ISGs) was also transcriptionally activated in *METTL1 KO* cells (Supplementary Fig. S7G). We also observed that transient silencing of *METTL1* in another cell line, 22Rv1, significantly increased the expression of ISGs genes, most likely due to a weak but not significant increased expression of IRF9 (Supplementary Fig. S7H, S7I). Given the anti-proliferative and pro-apoptotic effects of STAT1 activation [72, 73], we tested whether the growth defect in *METTL1 KO* PC3 cells could be reversed by downregulating *STAT1*. We found that apoptosis was reduced and proliferation was increased in PC3 *METTL1 KO* cells after *STAT1* knockdown (Fig. 6F, G), suggesting that the anti-proliferative effect of METTL1 inhibition can be in part mediated by activation of the STAT1 signalling pathway.

Taken together, our data suggest that METTL1-mediated tRNA methylation and tRNA fragment biogenesis induce a translational programme that activates the IFN signalling pathway. To confirm that high *METTL1* expression correlates with a decrease in IFN pathway activity in human PCa samples, we assessed ISGs expression in three PCa expression datasets [3, 6, 7]. We observed a significant inverse correlation between *METTL1* and *STAT1 expression* (Supplementary Fig. S7J). Similarly, we found an inverse correlation, although weaker, between *METTL1, IFIT1* and *IFIT2* expression (Supplementary Fig. S7J).

In summary, our data indicate that *METTL1* expression inversely correlates with activation of the IFN pathway in PCa cells and tumours, and METTL1 inhibition translationally activates the IFN signalling pathway.

### Low *METTL1* expression in PCa correlates with increased pro-inflammatory immune cell polarisation

**Fig. 7:**
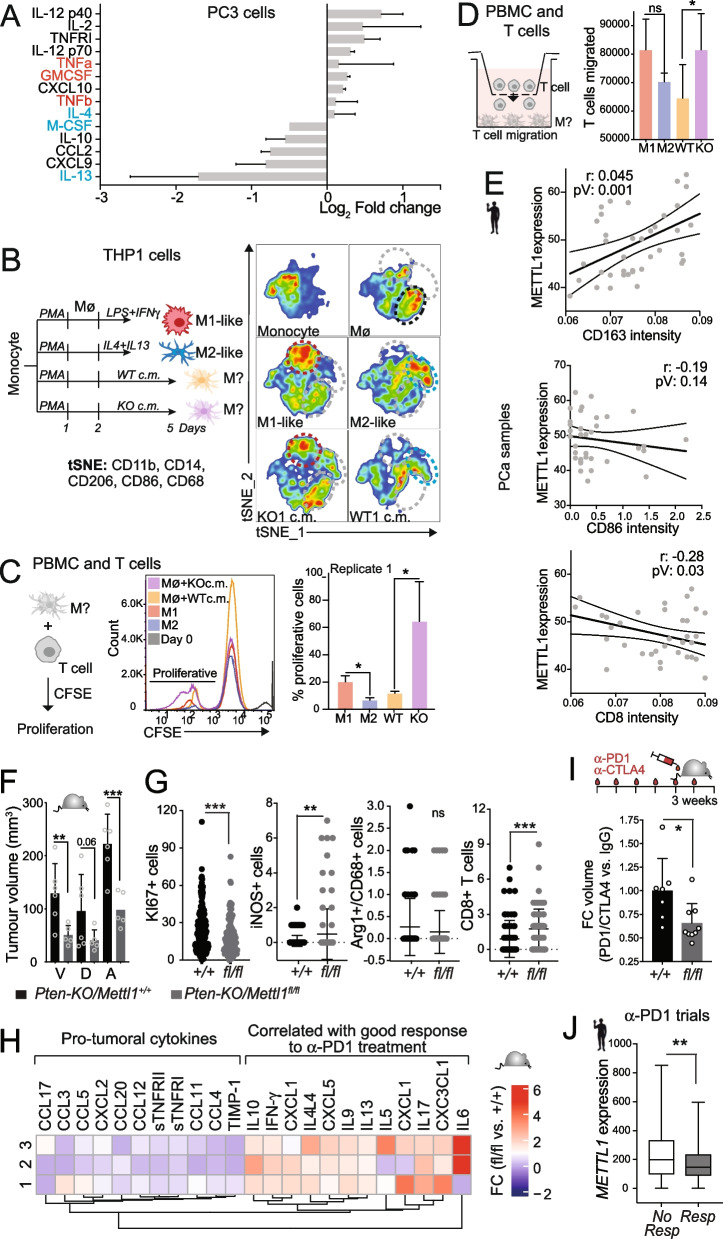
*METTL1* low expression in PCa is associated with increased cytotoxic infiltration and good response to ICB treatment. **A** Cytokine content in PC3 *METTL1 KO* compared to WT cell-conditioned media shows upregulation of pro-inflammatory (red) and downregulation of anti-inflammatory (blue) cytokines in *METTL1 KO* cells. The mean ± SD log_2_ fold change is shown (*n* = 4). The full expression array is shown in supplementary figure S8. **B** The upper panel shows a schematic overview of the workflow followed to analyse M1- or M2-like endotype polarisation of THP-1-derived Mø macrophages exposed to *METTL1 KO* and WT cell-conditioned medium (c.m.). The lower panel shows the T-distributed stochastic neighbour embedding (tSNE) analysis of macrophage polarisation (*n* = 3). **C** Proliferation of human peripheral blood CD3+ T cells co-cultured with primary Mø macrophages exposed to PC3 WT or *METTL1 KO* cells*’ c.m.* (n = three technical, two biological replicates). **D** Migration of human peripheral blood CD3+ T cells towards primary macrophages exposed to PC3 WT or *METTL1 KO* cells*’ c.m*. (n = three technical, two biological replicates). **E** Correlation between *METTL1* expression and the immune cell infiltrates of M1-like macrophages (CD86), M2-like macrophages (CD163), and CD8+ T cells in human PCa samples. Immunostainings are shown in supplementary figure S8. **F** Prostate tumour volume from *Pten-KO/Mettl1* + */* + and *Pten-KO/Mettl1*^*flox/flox*^ five-month-old mice reflects reduced tumour burden after conditional *Mettl1* deletion*.* Ventral (V), dorsal (D) and anterior (A) lobes. Mean ± SD, n ≥ 5. **G** Conditional deletion of *Mettl1* resulted in reduced tumour proliferation (Ki67 + cells) and increased immune infiltration of iNOS + (M1-like) macrophages and CD8+ T cells. Staining of tumours from *Pten-KO/Mettl1* + */* + (+ / +) and *Pten-KO/Mettl1*^*flox/flox*^* (fl/fl)* five-month-old mice*.* Mean ± SD, n ≥ 5, > 10 images per biological replicate. **H** Fold change of cytokines content in *Pten-KO/Mettl1*^*flox/flox*^ versus *Pten-KO/Mettl1* + */* + tumours (*n* = 3). **I** Significant decrease in tumour volume (fold change: FC) in *Pten-KO/Mettl1*^*flox/flox*^ (fl/fl) mice treated with anti-PD1 + anti-CTLA4 antibodies compared to untreated controls (IgG). *Pten-KO/Mettl1* + */* + (+ / +) mice tumour volume did not change after anti-PD1 + anti-CTLA4 treatment. Mean ± SD, n ≥ 6. **J** *METTL1* mRNA expression levels in anti-PD1 responders and non-responders in clinical trials of breast cancer, ovarian cancer, colorectal cancer, and glioblastoma (*n* = 484). The data were retrieved from the ROC plotter. Statistical tests: Pearson’s correlation (r), *p*-value (pV), and linear regression with 95% confidence (bands) are shown (**E)**. One-tailed Student’s t-test (**A, C, D, G**)**,** Mann–Whitney test (**F, I, J**), *p < 0.05, **p < 0.01, ***p < 0.001

Recently, epigenetic targeted therapies have been shown to trigger an IFN antiviral response in several cancer types, including PCa, mounting an innate immune response and resulting in the production of many cytokines [74–77]. To elucidate whether METTL1 inhibition can mount an innate immune response by activating the IFN signalling pathway, we investigated whether *METTL1* downregulation could alter cytokine expression and secretion in PCa cells. Overall, in *METTL1* KO cells, the cytokine composition analysis revealed an increased secretion of cytokines involved in pro-inflammatory activity and which polarise macrophages to M1-like endotype [78], including granulocyte–macrophage colony-stimulating factor (GM-CSF) and tumour necrosis factor α (TNF-α) (Fig. 7A; Supplementary Fig. S8A). *METTL1* removal also induced downregulation of anti-inflammatory cytokines, including Macrophage or Colony stimulating factor (M-CSF), IL10 and IL13 (Fig. 7A; Supplementary Fig. S8A), which can polarise macrophages to M2-like endotype [78]. These data suggested that METTL1 inhibition in tumour cells could polarise immune cells in the tumour microenvironment (TME) towards a cytotoxic tumoricidal endotype.

The advent of molecular technologies, such as single-cell RNA sequencing (scRNA-seq) and spatial transcriptomics, has enabled unprecedented auditing of the composition of the tumour immune microenvironment. Unsupervised clustering of scRNAseq and spatial transcriptomics analysis has revealed that within the innate immune population, macrophages constitute the largest proportion in PCa [79–81]. Tumour-associated macrophages (TAMs), which typically exhibit an expression pattern characteristic of M2-like or pro-tumoural macrophages [82], represent a major component of the TME supporting tumorigenesis [83]. They are known to facilitate cancer cell proliferation, invasiveness, angiogenesis, and immunotolerance, while also contributing to resistance against standard-of-care therapies, immunotherapies, and chemotherapy [84–86]. Consequently, there is a critical need to develop clinical agents that can either deplete macrophages or induce their repolarisation toward an M1-like or anti-tumorigenic state [84, 87].

Taking these circumstances into account, along with the observation that METTL1-expressing cells secrete cytokines known to drive macrophage polarisation towards the M2-like endotype, we focused on elucidating the role of METTL1 in macrophage polarisation. We incubated the human monocyte cell line THP1 with conditioned media (c.m.) from PC3 WT or *METTL1 KO* cells and investigated the expression of M1-like and M2-like macrophage endotype markers (Fig. 7B). tSNE analysis showed that while c.m. from WT cells induced macrophages towards an M2-like endotype, inhibition of *METTL1* in PCa cells induced macrophages towards an M1-like endotype (Fig. 7B; Supplementary Fig. S8B). Macrophages in the presence of *METTL1 KO* cells’ c.m. also showed a phagocytic capacity similar to that of M1-like macrophages (Supplementary Fig. S8C). Similar results were obtained when primary peripheral blood monocytes were exposed to PC3 *METTL1 KO* cells’ c.m. (Supplementary Fig. S8D). In addition, peripheral blood macrophages cultured in the presence of PC3 *METTL1 KO* cells’ c.m. showed higher induction of the proliferation and migration of CD3+ T cells (Fig. 7C, D; Supplementary Fig. S8E). Altogether, our data suggested that *METTL1* inhibition in cancer cells may stimulate a cytotoxic and anti-tumoural inflammatory response in the TME.

To validate our findings in vivo, we evaluated the intratumoural immune cell composition of human prostate tumours using immunohistochemical analyses. The analysis showed a significant direct correlation between M2-like macrophage infiltration and METTL1 expression at the expenses of an inverse trend in M1-like macrophage infiltration in PCa paraffin samples. Similarly, CD8+ T cells infiltration correlated inversely with METTL1 expression (Fig. 7E; Supplementary Fig. S8F). Additionally, deconvolution analysis of PCa expression datasets using CIBERSORT [88] or TIMER [89] showed a significant inverse correlation between intratumoural M1-like macrophage composition and *METTL1*, indicating a more cytotoxic immune tumour component in METTL1-low tumours (Supplementary Fig. S8G, S8H).

Altogether, our results demonstrated that inhibition of *METTL1* expression in PCa cells can mount a cytotoxic immune response in vitro and further support that low expression of *METTL1* in human PCa is associated with an increase in intratumoural cytotoxic immune cells in the TME.

### *Mettl1* inhibition enhances the response to immune checkpoint blockade therapy in PCa

To assess whether *METTL1* inhibition could reduce the tumour growth and affect the intratumoural immune composition in vivo, we deleted *Mettl1* in *Pten-KO* mice (Supplementary Fig. S9A, S9B). *Mettl1* systemic homozygous deletion was lethal (data not shown). *Mettl1* heterozygous deletion (*Pten-KO/Mettl1* +/-*)* and *Mettl1* conditional deletion in the prostatic epithelium of *Pten*-KO mice (*Pten-KO/Mettl1*^*flox/flox*^*)* resulted in a significant decrease in the tumour volume of the ventral and anterior lobes, which were consistently larger in *Pten-KO/Mettl1* + */* + mice (Fig. 7F; Supplementary Fig. S9F). Systemic and conditional knockdown of *Mettl1* in *Pten-KO* mice significantly increased the formation of 5′tRNA fragments, indicating that tRNA cleavage protection by m^7^G methylation is a conserved mechanism in mice (Supplementary Fig. S9C). Histological analyses of tumours showed that, while *Mettl1*-expressing tumours were invasive and consisted of undifferentiated cells, tumours from *Pten-KO/Mettl1* +/- and *Pten-KO/Mettl1*^*flox/flox*^ mice were encapsulated and consisted of differentiated luminal cells with low proliferative potential (Supplementary Fig. S9D, S9E, S9G), suggesting that *Mettl1* is required for tumorigenesis.

To investigate whether the mechanism underlying the anti-tumour effect of *Mettl1* deletion was due to tumour cell intrinsic pathways or to the tumour immune microenvironment, we injected tumour cells isolated from *Pten-KO/Mettl1* + */* + and *Pten-KO/Mettl1*^*fl/fl*^ mouse prostates into NOD scid gamma (NSG) immune-incompetent mice. Allografts growth revealed a slight but non-significant reduction in the growth of allografts derived from *Pten-KO/Mettl1*^*fl/fl*^ mice (Supplementary Fig. S9H). These findings provided important insights into the mechanism underlying the anti-tumour effect of *Mettl1* deletion and suggested that other than tumour cell intrinsic pathways were involved in the anti-tumour effects observed upon *Mettl1* deletion in vivo.

Based on these observations and the capability of *METTL1*-deficient cells to elicit a cytotoxic immune response characterised by the induction of M1-like macrophage polarisation, as well as enhanced proliferation and migration of CD8+ T cells in vitro (Fig. 7A-D, Supplementary Fig. S8A-E), our subsequent investigation focused on analysing the composition of macrophages and CD8+ T cells in *Mettl1*-deficient prostate tumours. Immunohistochemical analysis revealed a significant increase in intratumoural infiltration of M1-like macrophages (iNOS+) at the expense of M2-like macrophages (Arg1/CD68+), and an increase in CD8 + T cells in *Mettl1* +/- and *Mettl1*^*flox/flox*^ tumours compared to *Mettl1*^+*/*+^ tumours (Fig. 7G; Supplementary Fig. S9E, S9G).

Cytotoxic T cells, distinguished by their expression of CD8, possess the remarkable ability to directly recognise and eliminate cancer cells, making them the most potent effectors in the anticancer immune response [90]. To determine whether the observed anti-tumour effect upon *Mettl1* deletion in mice was due to increased intratumoural infiltration of cytotoxic CD8+ T cells, we examined the effect of systemic elimination of CD8+ T cells in *Pten-KO/Mettl1* + */* + and *Pten-KO/Mettl1*^*fl/fl*^ mice treated with anti-CD8α antibodies. Our results showed that the elimination of CD8+ T cells led to a significant increase in the size of only anterior lobe tumours lacking *Mettl1* (Supplementary Fig. S9I). These results suggested the partial involvement of CD8+ T cell infiltration in the anti-tumour effect observed in *Mettl1*-deficient tumours, however, it also indicated the involvement of alternative immune cell types or pathways.

Cancer-related inflammation plays a crucial role in the progression of most cancer types. Components of both the innate and the adaptive immune system that are recruited upon tumour onset interact with each other and with the tumour, thus building an immune response in situ. In prostate tumours, various types of cytotoxic immune cells have been observed to infiltrate the TME [79, 81]. Dendritic cells (DCs), are crucial antigen presenting cells that present MHC-peptides to cytotoxic T cells, play a vital role in tumour immunotherapy [91]. Natural killer (NK) cells are an innate lymphoid cell with cytotoxic function and can directly kill tumour cells without prior sensitisation [92]. The adaptive immune system, composed of CD8+ T and also CD4+ T cells, and B cells [79], plays a pivotal role in mounting an effective, antigen-specific immune response against tumours. Cytotoxic T cells for instance are key effectors of the adaptive immune system and can directly recognize and eliminate cancer cells [90]. Substantial evidence has shown that cancer cells can orchestrate this pro-tumourigenic response by reprogramming the immune system. Thus, future functional validations showing how *METTL1*-expressing tumour cells interact with their neighbouring immune cells will lead to a better understanding of PCa progression.

To elucidate the impact of *Mettl1* inhibition on the reprogramming of the immune tumour composition, we employed an analysis of immunomodulatory molecules, including cytokines and chemokines, as surrogate markers of the immune cells present in the tumour. Our results revealed a significant shift in the cytokine composition, characterised by a decrease in the secretion of pro-tumoural cytokines. Additionally, we observed an increased secretion of cytokines known to correlate with a favourable response to immune checkpoint blockade (ICB) therapy, in *Pten-KO/Mettl1*^*fl/fl*^ tumours (Fig. 7H) [93]. These findings suggest that Mettl1 inhibition may contribute to an immune microenvironment conducive to improved ICB therapy outcomes, highlighting the potential of targeting Mettl1 as a therapeutic strategy for enhancing the anti-tumour immune response.

To determine if the cytokine intratumoural composition in *Mettl1*-deficient tumours could enhance the efficacy of ICB therapy, we treated mice with anti-PD1 and anti-CTL4A antibodies. *Pten-KO/Mettl1* + */* + mice did not shown tumour volume reduction after ICB treatment compared with that in untreated mice, whereas *Pten-KO/Mettl1*^*flox/flox*^ mice exhibited a significant reduction in tumour volume after ICB treatment (F ig. 7I; Supplementary Fig. S9J). Remarkably, the tumour volume of *Pten-KO/Mettl1*^*flox/flox*^ mice after ICB treatment was reduced to almost the size of the normal prostate (Supplementary Fig. S9J). Therefore, the results strongly suggested that decreased expression of *Mettl1* increased the efficacy of ICB therapy in preclinical mouse models.

We also investigated whether *METTL1* expression levels could predict the efficacy of ICB therapy in patients. Using the ROC plotter platform, we analysed the expression levels of *METTL*1 in several tumour types treated with anti-PD1 therapy [94]. We found that *METTL1* expression was lower in responders than in non-responders to ICB therapy in breast, colorectal, ovarian cancer, and glioblastoma patients, suggesting that high *METTL1* expression predicted poor response to ICB therapy (Fig. 7J).

In summary, our findings demonstrate that *METTL1* expression levels can determine intratumoural cytotoxic immune infiltration and the success of ICB therapy.

## Discussion

RNA modifications are emerging as important modulators of biological processes and pathological progression. Although recent studies have shown that their dysregulation is associated with cancer, we are in the infancy of our understanding of the molecular basis. In particular, tRNA modifications are emerging as important regulators of cancer; however, molecular insights remain poorly understood [95]. In this regard, our study identifies that increased expression of the tRNA methyltransferase METTL1 and elevated 7-methylguanosine deposition in tRNAs have oncogenic potential by altering the translational programme in cancer cells. In line with our findings, recent evidence has associated increased *METTL1* expression with the occurrence of several cancer types [22, 23, 28, 29, 32–34, 36, 37, 96, 97]. In addition, we found that increased expression of *Mettl1* correlates with increased m^7^G deposition in tRNAs and increased levels of excreted 7-methylguanine in the urine in mouse PCa. Increased m^7^G excretion has been found in other tumour-bearing rodents [98] and in patients with cancer, including PCa [99–102]. This evidence suggests that both *METTL1* expression and m^7^G deposition levels may serve as potential tumour biomarkers [103].

PTEN-PI3K pathway is among the most commonly altered pathways in advanced PCa and is a critical indicator of disease progression in hormone-naive and castration-resistant prostate cancer (CRPC) [47]. Thus, mechanistic understanding and identification of the downstream effectors of the PI3K-PTEN oncogenic signalling pathway can lead to better patient stratification and the design of better therapeutic strategies. Here, we provide unprecedented evidence of the control of tRNA methylation downstream of the AKT pathway through the control of *METTL1* expression. Consistent with our findings, METTL1 was reported to be regulated by AKT and ribosomal S6 kinase in embryonic stem cells [104]. In hepatocellular carcinoma, *METTL1* expression has been associated with PTEN signalling [33]. Moreover, in lung adenocarcinoma cell lines, a positive feedback loop has been suggested, in which *METTL1* expression enhances the AKT-mTOR signalling pathway [97]. These findings highlight the complex regulatory landscape that controls tRNA modifications and integrates and translates growth signals into oncogenic epitranscriptomic programmes.

Our study provides strong evidence supporting an oncogenic role for METTL1-mediated tRNA methylation in PCa. We found that the deletion of *METTL1* dramatically suppressed tumorigenesis both in vitro and in vivo. In contrast, re-expression of only a catalytically active version of METTL1 promotes the proliferation of cancer cells, suggesting that METTL1-mediated RNA methylation has oncogenic potential. Consistent with our functional in vitro and in vivo data on PCa, recent studies have highlighted the oncogenic role of METTL1 in several cancer types [22, 23, 28, 33, 37, 97]. Mechanistically, we found that METTL1 primarily methylates tRNAs to protect them from endonucleolytic cleavage, thereby determining the production and identity of a subset of tRNA fragments (tRFs) known as 5′TOGs. The production of tRFs is a common response to various stimuli, including stress, and their biogenesis is regulated by RNA modifications [58]. In this regard, tRNA fragment diversity and abundance are particularly associated with cancer [105], with PCa showing the highest repertoire [106, 107]. The molecular role of tRNA cleavage is not to degrade hypomodified tRNAs; instead, 5′TOGs are specifically produced to reprogramme mRNA translation [16, 55, 57, 60, 67]. Here, we found that in *METTL1*-deficient cells, 5′TOGs displace translation initiation factors from capped-mRNAs to favour the translation of a distinct set of genes involved in tumorigenesis and stress responses. Recent studies have shown that METTL1 regulates self-renewal, differentiation, and growth through different mechanisms related to codon-specific translational control [22, 23, 27, 28]. Thus, our study uncovered new layers of selective regulation of gene expression that cancer cells selectively utilize to favour survival and growth, independent of transcriptional control.

Protein synthesis rates and translational programmes are highly regulated in homeostasis, and dysregulation of these translation programmes is associated with cancer [108, 109]. In PCa, the PI3K-mTOR axis steers cancer pathogenesis by regulating alternative translation initiation factors that favour distinct translational programmes [110]. In this regard, our findings support a model whereby tRFs biogenesis is controlled by epitranscriptomic regulation to reprogramme the translatome to allow cell growth and adaptation to the TME [16, 55, 57, 59].

In our study, we have identified METTL1, by modulating mRNA translation, is a crucial regulator of the IFN-STAT1 signalling pathway in tumour cells, implying its significance in prostate cancer and the tumour microenvironment. Our findings indicate that inhibiting METTL1 activity leads to favourable changes within the tumour, such as an increased presence of anti-tumoural cytokines and the infiltration of cytotoxic immune cells, including M1-like macrophages and CD8+ T cells. The results of our research provide compelling evidence highlighting the pivotal role of METTL1 in regulating the immune response against tumours. This emphasises its potential as a promising target for novel cancer therapies, either as a standalone treatment or in combination with immune checkpoint blockade therapy. In line with our observations, recent studies have shown that METTL1 is involved in mediating the mRNA translation of cytokines that induce the accumulation of myeloid-derived suppressor cells (MDSCs) in intrahepatic cholangiocarcinoma [111], a major immune suppressive subset infiltrating these tumours [112]. Moreover, our study aligns with previous evidence demonstrating the association between METTL1 dysfunction and chronic immune conditions in humans, such as psoriasis and multiple sclerosis [113–116]. It will be interesting in future studies to explore the immune landscape in the context of METTL1 to gain a more comprehensive understanding of the implications for tumour immune evasion in PCa other cancer types. In conclusion, our study underscores the critical role of METTL1 in PCa and the tumour microenvironment. The insights gained from this research open up new possibilities for targeted therapies that modulate METTL1 activity, which may enhance the anti-tumour immune response and improve patient outcomes in PCa. These findings also highlight that the impact of METTL1 extends beyond cancer and may have implications in broader immune-related disorders.

Central to the efficacy of ICB therapy is the requirement for cytotoxic immune cells to infiltrate the tumours [117]. ICB therapy generates durable therapeutic responses in a significant subset of patients across a variety of cancer types [118]. However, PCa has shown resistance to ICB owing the high infiltration of immunosuppressive myeloid cells [79–81, 119–122]. In addition, suppression of the tumour-intrinsic IFN pathway is strongly associated with tumour cell outgrowth and metastasis in PCa [123]. Emerging evidence has demonstrated that enhancing immune responses and reprogramming or re-education of intratumoural immune cells towards a tumoricidal endotype is an effective strategy to boost ICB therapy to treat cancer [124–126]. Epigenetic inhibitors are emerging as immune modulators of the TME. Epigenetic inhibitors can induce IFN pathway activation [74, 127], re-educate TAMs towards an M1-like endotype, and increase intratumoural cytotoxic T cell infiltration, all resulting in superior immune therapeutic benefits when combined with ICB [75, 128–132]. The therapeutic potential of targeting the epitranscriptome in cancer remains largely unexplored, but recent research suggests that the epitranscriptome may play a role in immune regulation [133–135]. Our study contributes to this understanding by demonstrating that inhibiting METTL1 leads to enhanced infiltration of cytotoxic macrophages and cytotoxic T cells into tumours, indicating that METTL1 serves as a key mediator of intratumoural immune responses. These findings suggest that combining METTL1 inhibitors with immunotherapy could have a synergistic effect, increasing the efficacy of cancer treatment.

## Conclusions

Our study provides compelling evidence of METTL1's oncogenic function and overexpression in prostate cancer (PCa). We also discovered a new layer of expression regulation and selective translation control through tRNA fragment biogenesis. By inhibiting METTL1-mediated methylation, we were able to increase the biogenesis of a novel class of ncRNAs (5'TOGs), which regulate the activation of the interferon signalling pathway in cancer cells. Additionally, METTL1 inhibition in cancer cells leads to increased infiltration of cytotoxic immune cells, which transforms the immunosuppressive prostate TME into a tumoricidal endotype. These results suggest that targeting METTL1 alone or in combination with immune checkpoint inhibitors holds great potential for developing effective therapeutic strategies, particularly for patients with castration-resistant prostate cancer who have limited treatment options.

## Experimental procedures

### Cell culture

Human prostate cell lines and prostate cancer cell lines PC3, DU145, VCaP, C4-2, 22Rv1, LNCap, BPH1, RWPE-1, PWR-1E, and HEK293FT were from DSMZ, ATCC and ThermoFisher Scientific and provided by Pr. A. Carracedo (CIC-bioGUNE, Spain). THP-1 cells were kindly provided by Dr. M. Fuentes García (CIC, Spain). PC3, DU145, VCaP and HEK293FT were cultured in DMEM with L-glutamine and pyruvate (Gibco) supplemented with 10% fetal bolvine serum (FBS (Gibco) and 1% penicillin/streptomycin. THP-1, 22Rv1, and LNCaP were cultured in RPMI with L-glutamine and pyruvate (Gibco) supplemented with 10% FBS (Gibco) and 1% penicillin/streptomycin. BPH1 cells were cultured in RPMI with 20% FBS, 20 ng/mL testosterone, 5 µg/mL transferrin, 5 ng/mL sodium selenite, and 5 µg/mL insulin. PWR-1E and RWPE-1 cells were grown in keratinocyte serum-free medium (K-SFM) (Gibco) supplemented with 0.05 mg/ml Bovine Pituitary Extract (BPE, Gibco) and 5 ng/ml Endothelial Growth Factor (EGF, Gibco). All the cells were maintained in a humidified atmosphere at 37 °C with 5% CO_2_. Cell cultures were tested for mycoplasma monthly and maintained mycoplasma-free. Peripheral blood mononuclear cells (PBMC) from healthy donors were provided by Biobanco del Centro de Hemoterapia y Hemodonación de Castilla y León. All donors were males, older than 50 years, and without prostate cancer. PBMC were cultured in RPMI with L-glutamine and pyruvate (Gibco) supplemented with 10% FBS (Gibco), 1 mM sodium pyruvate (Gibco), 10 µM HEPES (Gibco), and 1% penicillin/Streptomycin.

### PI3K pathway inhibition and DHT treatment

PC3 and DU145 cells were grown in complete medium and treated with rapamycin (LC Laboratories), Torin-1 (Tocris), MK2206 (Selleckchem), and BKM120 (Selleckchem). All were prepared in DMSO and used at a final concentration of 20 nM (Rapamycin), 125-250 nM (Torin-1), 5 μM (MK2206) and 100 nM BKM120, for the indicated times in each experiment. Two technical replicates were performed for each experiment, experiments were repeated three times. LNCaP cells were grow in RPMI with L-glutamine and pyruvate (Gibco) supplemented with 10% FBS (Gibco) and 1% penicillin/streptomycin and were treated for 6 h with dihydrotestosterone (DHT) (Sigma) at 10 nM.

### Mouse lines

All mice were housed at the Animal Research Core Facility at CIC-bioGUNE and at the University of Salamanca and maintained in ventilated filter cages under specific pathogen-free conditions with food and water available ad libitum. All mouse experiments were carried out following the ethical guidelines established by the Biosafety and Bioethics Committee at CIC bioGUNE (under protocol P-CBG-CBBA-0715), and at the University of Salamanca (under protocols #269, #506, #595, #707), and by the Competent Authority of the Basque Country and Castilla y León. Xenograft experiments were performed by subcutaneously injecting 1 × 10^6^ PC3 WT and METTL1 KO cells with Matrigel (BD Biosciences) in two flanks per mouse of eight weeks old athymic nude mice (Envigo). pLKO–Tet-on-shRNA-WDR4-2 or pLKO–Tet-on-shRNA-SCR cells were injected into each flank of athymic nude mice and when tumours were growing, mice were fed normal or doxycycline diet (Research diets, D12100402) to induce silencing. Mice in this group were left on continuous doxycycline diet. Cells were pre-treated for 48 h with PBS or doxycycline (0.1–0.5 μg/ml). Allograft experiments were performed by injecting 1 × 10^6^ tumour cells isolated from five months old *Pten-KO/Mettl1* + */* + and *Pten-KO/Mettl1*^*flox/flox*^ mice. To isolate cells from *Pten-KO/Mettl1* + */* + and *Pten-KO/Mettl1*^*flox/flox*^ mice, tumours were dissected and dissociated for 1 h at 37ºC in rotation with DMEM-F12 medium containing 5% FBS and 1 × collagenase Type 1 (ThermoFisher) and 1 × hyaluronidase (Stem Cell Tech.). Next, cells were incubated for 1 h at 37ºC with 0.25% trypsin, followed by incubation with 5 mg/ml Dispase II (Sigma-Aldrich) and 1 U/µl DNase (Sigma-Aldrich) for 30 min at 37ºC. Dissociated cells were then filtered through a 0.45 µm cell strainer and mixed 1:1 with Matrigel (BD Biosciences) and injected subcutaneously into both flanks of NSG (NOD.Cg-Prkdc^scid^ Il2rg^tm1Wjil^/SzJ) mice (Charles River). Tumour were grown to a maximum total diameter of 1.2 cm of diameter. Tumour volume was estimated using the following formula: volume = (length x width)^2^ × 0.526.

To generate a conditional and straight *Mettl1* mouse model, we floxed exon 2 of *Mettl1* at a residue overlapping the splicing site (Supplementary Fig. S9A). In silico analysis revealed that an early truncated METTL1 protein without catalytic activity was produced when exon 2 was removed. Two complementary RNA oligos corresponding to crRNAs (CRISPR RNA), named METTL1 crRNA guide 1 and guide 2 (Mettl1 crRNA guide1: rU rA rA rG rA rG rC rC rA rU rG rA rU rG rA rUr C rC rA rA rG rU rU rU rU rA rG rA rG rC rU rA rU rG rC rU; Mettl1 crRNA guide2: rU rG rG rC rU rU rG rU rU rA rG rG rU rA rA rU rA rA rG rC rG rU rU rU rU rA rG rA rG rC rU rA rU rG rC rU), were designed with the Spanish National Biotechnology Centre (CNB)-CSIC web tool (http://bioinfogp.cnb.csic.es/tools/breakingcas/). These crRNAs contained the target-specific sequence for guiding Cas9 protein to *Mettl1* exon 2 and the adjacent intron sequence. An 872nt single-strand DNA (METTL1 ssODN: CGGGTAAATAAAAATTTTAAAATATACATAAACAAAGTAGTGCTCCAGTGCCTTAGGCAGTCAGTCTCTGGGAAAACCTTACACACATCAGTACCCAGAAGAACTTGGCAGATCTGCAGCCATAGCCTCATGATGGGACTGATTCACACCCTAGAAGCCAATAACTTCGTATAATGTATGCTATACGAAGTTATGGGGACTTCCCATTACACCTTCTACATACCACATGGTCCCTGCATGCAGAGTTTTTTTCTAAGCCTACCACCCCACCCCCAACTCTTACACAAAATGTCTGCACTGGTATGTACAACAGTCTCATGTGTCTCATGTCTTCTTTCTAATAGCCCTGTGAAGCCAGAGGAAATGGACTGGTCTGAGCTTTACCCAGAGTTCTTTGCTCCGCTTATTCAAAATAAGAGCCATGATGATCCAAAAGATGAGAAAGAAAAGCACTCTGGGGCCCAAGTGGAGTTTGCAGACATAGGCTGTGGCTATGGTGGCTTGTTAGGTAATAAGCTCGCCCTTTTCTTGGGACAGGGAGAGGCCTGGGTTCTGCCATCTCAGCAGGTTAGAGGCAGGATTAGTTGACCTTTCCTCCTGGGACCAGACAGCAGTGACATCAGTGTGGAGAGCCTCCACCTTCCTTCTACTCTGGGTCATAACTTCGTATAATGTATGCTATACGAAGTTATTGTGGCCTTTGCCCTGGAGGAGGGAGGGGGCTGCCTCAGATGTATCTGAGAACCCCGGTTGTTCTGTCTAGCTCCAGTGGCTCCAGTCCCTCCGTGAGCCATTCCACTGCCTTGTGCTGGGTCAGTGAGCCGGGTCAGCACAAGCGTCAGAGATCAGCTCCAGTGGCCACTGATCCTGAA) was designed as a template for homologous recombination. The METTL1 ssODN donor DNA sequence contained two LoxP sequences in the same orientation flanking exon 2 and two single mutations destroying the PAM sequences. The crRNAs, ssODN, and trans-activating CRISPR RNA (tracrRNA) were produced by chemical synthesis at Integrated DNA Technologies (IDT). The crRNA and tracrRNA were annealed to obtain the mature sgRNAs. A mixture containing sgRNAs, recombinant Cas9 protein (IDT), ssODN [Cas9 (30 ng/µL), annealed sgRNA (20 ng/µL each), and ssODN (10 ng/µL)] was microinjected into C57BL6/J zygotes at the Transgenic Facility (NUCLEUS, platform for research support of the University of Salamanca). All edited founders were identified by polymerase chain reaction (PCR) amplification (Taq polymerase, NZYTech) with primers flanking exon 2 (primer F 5’-TCTCCTGGTGTGCATGAAGAC-3’ and R 5′-AGGGAAGGTGGTGGAATCCC-3′), producing an amplicon of 957 base pairs (bp) for edited alleles with a new enzyme restriction site. PCR products from the founders were subcloned into pBlueScript (Stratagene) and confirmed by Sanger sequencing. The founders were crossed with WT C57BL/6 J (produced at NUCLEUS, platform for research support of the University of Salamanca) to eliminate possible unwanted off-targets. LoxP-edited (Mettl1-flox allele) or mice with point mutations/deletions at exon 2 (Mettl1 ± allele) were obtained. Heterozygous mice were re-sequenced and crossed to give rise to edited heterozygous and homozygous mice. Genotyping was performed by PCR with primers F-R and Restriction Fragment Length Polymorphism (RLFP) analysis (1F: 5’-TCTCCTGGTGTGCATGAAGAC-3’; 1R: 5’- AGGGAAGGTGGTGGAATCCC -3’. To assess *Mettl1* status in established mouse colonies, genotyping was performed by PCR using primers F-5’-ATCTGCAGCCATAGCCTCAT-3’ and R-5’-TCTAACCTGCTGAGATGGCA-3’, for floxed alleles, followed by a digestion with Bcl-I restriction enzyme (New England Biolabs) for straight *Mettl1* ± mice since CRISPR editing caused a point mutation generating a Bcl-I restriction site. We did not obtain *Mettl1-/-* mice*,* suggesting the lethality of the homozygous allele. qPCR confirmed decreased expression of *Mettl1* in all tested tissues from *Mettl1* ± , or in the prostate of *Probasine-Cre* *x* *Pten* ^*flox/flox*^ *x* *Mettl1* ^*flox/flox*^ mice.

The *Pten*^*flox*^ conditional knockout allele and the prostate epithelium-specific Cre recombinase Pb-Cre4 have been described elsewhere [45] and were provided by Pr P. P. Pandolfi. For prostate tumour evaluation upon *Mettl1* deletion, *Mettl1* ± *or Mettl1* ^*flox/flox*^ mice were crossed to *Pten *^*flox/flox*^ and *Pb-Cre* mice.

### Patient samples

RNA and protein samples from prostate specimens were obtained upon informed consent and with evaluation and approval from the corresponding ethics committee (CEIC codes OHEUN11-12 and OHEUN14-14), as described elsewhere [136].

### Computational expression and mutation load analysis from human prostate tumour datasets

For expression analyses of human prostate tumours in different disease status or recurrence, we combined the use of CANCERTOOL [137] with complementary ad hoc analyses on various datasets [3, 6, 7, 42–44, 138]. All downloaded data were pre-processed, background corrected, and log_2_ transformed and quantile normalised according to [137]. Differential expression was computed by means of Z-scores as the number of standard deviations of expression of cancerous samples relative to the mean of expression in a reference population (primary tumour vs. normal and metastasis vs. normal). Subsequently, an ANOVA test was performed for the different groups. Finally, we calculated coherence as the percentage of datasets in which the gene was significant out of the number of datasets in which the gene appeared (ANOVA *P*-value significant ANOVA (value < 0.05)). Only genes with consistent differential expression in at least three datasets and 75% coherence were considered significant. Correlation analyses (Pearson’s correlations) and data [3, 6, 7, 42, 138] were retrieved from CANCERTOOL [137].

For disease-free survival, expression data were organised into deciles for (TCGA) [3]. The Cox proportional hazards model was used to evaluate the association between the survival time of patients and expression levels of primary tumours. For biochemical recurrence-free survival of patient groups from the Cambridge and Stockholm cohorts, data were retrieved from camcAPP [48].

For correlation analysis, data were retrieved from CANCERTOOL [137].

### Cloning and generation of stable cell lines

For lentiviral particle production, HEK293FT cells were transfected with calcium phosphate or JetPEI (PolyPlus) using the appropriate expression vector. Cell transfection with lentiviral vectors was performed according to the standard procedures. Briefly, viral supernatants from 48 and 72 h post-transfection were collected and concentrated using a Lenti-X concentrator (Takara). Lentiviral particles were resuspended in 200 μL of complete medium and added to 1 × 10^6^ cells in medium supplemented with 8 μg/mL Protamine Sulfate (Sigma). Transduced cells were selected using 2 μg/mL puromycin (Sigma) or 10 μg/mL blasticidin (Santa Cruz Biotechnology) for 3 or 5 days, depending on the selection cassette.

For CRISPR-Cas9 editing of the *METTL1* coding region, two single-guide RNAs (sgRNAs) targeting exons 2 and 3 of *METTL1* were designed using the *crispor* software (http://crispor.tefor.net/). sgRNAs (sg117-5’-TATGTCTGCAAACTCCACTTGGG-3’ and sg139: 5’-CAAGTGGAGTTTGCAGACATAGG-3’) were cloned into the lentiviral vector LentiCRISPR-v2 (Addgene, 83,480). As a control, LentiCRISPR-v2 expressing Cas9 was transduced into the cells. After cloning, plasmids were sequenced for further validation. Editing was performed in the PC3 and DU145 cell lines. Homogenous gene editing was ensured by generating single-cell clones for each sgRNA. Three clones were selected from each cell line. Knockout efficiency was tested by genomic DNA PCR amplification (with primers flanking the targeted region F: 5’-GTTCTTCGCTCCACTCACTC-3’ and R: 5’-CAAAAAGAGGGCCTGAGTGTT-3’) and Sanger sequencing. RT-qPCR and western blotting were also performed. All clones were validated by sequencing.

For inducible silencing of human *WDR4*, two different short-hairping RNA (shRNAs) sequences targeting human *WDR4* were cloned into the doxycycline-inducible lentiviral plasmid pLKO.1-TetON (Addgene, 21915). The shRNAs sequences used were: shWDR4-1: GCTGTTGAAACTCAGCACGAA; shWDR4-2: GCACCTGTCTATGCTGTTAGA and introduced *into Age*I and *Eco*RI restriction sites. A scramble control vector was generated by cloning oligo CCGGCAACAAGATGAAGAGCACCAACTCGAGTTGGTGCTCTTCATCTTGTTG. All clones were sequence-validated. Cells were infected with plasmids expressing either scramble control or shRNAs. shRNAs expression was pre-induced for 3 days with 0.1 µg/mL doxycycline (Sigma) and then induced for the experiment length at 0.25 μg/mL or directly induced with 0.5 μg/mL for 3–5 days. The silencing efficiency was tested both by RT-qPCR and western blotting.

HA-METTL1 expressing vector was generated starting from the ORF obtained from Source BioScience (clone: X96698; IMAGE clone: 36006) and cloned into a modified retroviral doxycycline-inducible pTRIPZ vector (Dharmacon) [139] with an HA-tag. Site-directed mutagenesis for the generation of METTL1-AFPA was performed using the Q5 Site-Directed Mutagenesis Kit (NEB) and the primers F: 5’-CCCGCCCCACATTTCAAGCGGACA-3’ and R: 5’-GAAGGCGAAGAACATCTTTGTCAGC-3’. PC3 parental lines and PC3 *METTL1 KO* cell lines were transduced with lentiviral constructs containing TRIPZ-HA-METTL1 (wild-type), TRIPZ-HA-METTL1-AFPA, or empty vector. Expression of the tagged version was confirmed by western blotting. Wild-type or mutant METTL1 expression was induced with 0.5 µg/mL doxycycline for 5 days for dot blot analysis and 0.1 µg/ml doxycycline for growth curve assays.

### Western blotting

For protein extraction from cultured cells, cells were scratched with lysis buffer (150 mM NaCl, 40 mM Tris pH 7,6; 1% Triton X-100, 1 mM EDTA, 1 mM MgCl2) supplemented with cOmPlete EDTA-free protease inhibitor cocktail (Roche) and phosphatase inhibitors (1 mM Sodium Fluoride, 1 mM Sodium Orthovanadate and 1 mM ß-glycerophosphate). To extract proteins from mouse tissue, samples were snap-frozen in liquid nitrogen and transferred to lysis buffer (without detergents) in a homogenization tube with ceramic beads. Tissue was homogenised using a FastPrep-2 5G Instrument (MP Biomedicals™), with two cycles of 30’’ at 6 m/s and detergents were added after that. The proteins were cleared by centrifugation for 10 min at 13,000 rpm. The total protein was quantified using the Pierce BCA Protein Assay Kit (Thermo Scientific). Equal amounts of protein were loaded onto homemade SDS-PAGE gels and then transferred onto nitrocellulose membranes (HE Healthcare) via wet transfer. Membranes were blocked with TBS-T 5% skimmed milk and primary antibodies were prepared in blocking solution. The following primary antibodies were used for western blotting: anti-METTL1 (Abcam, Ab157997), anti-WDR4 (Abcam, ab169526), anti-pS6K (Cell Signaling, 9205S), anti-S6K (Cell Signaling, 9202), anti-PTEN (Cell Signaling, 9559), anti-mouse AR (Cell Signaling, 5153), anti-human AR (Abcam, ab133273), anti-pS6 (Cell Signaling, 4858), anti-S6 (Santa Cruz, sc-74459), anti-pAKT (Cell Signaling, 4060), anti-AKT (Cell Signaling, 9272S), anti-p4EBP1 (Cell Signaling, 2855L), anti-4EBP1 (Cell Signaling, 9644S), anti-HA (Biolegend, 901514), anti-peIF2α (Cell Signaling, 3597), anti-eIF2α (Cell Signaling, 5324), anti-eIF4AI/II (Santa Cruz, sc-377315), anti-eIF4E (Santa Cruz Biotechnology, sc-9976), anti-eIF4G, (Santa Cruz Biotechnology, sc-133155), anti-YB1 (Santa Cruz, sc101198), anti-PABP1 (Cell Signaling, 4992), anti-ISG15 (Santa Cruz Biotechnology, sc-69701), anti-IRF9 (Santa Cruz Biotechnology, sc-365893), anti-pSTAT1 (Santa Cruz Biotechnology, sc-8394), anti-STAT1 (Santa Cruz Biotechnology, sc- 417), anti-tubulin (Abcam, Ab15246), anti-GAPDH (Cell Signaling, 2118) and anti-HSP90 (Santa Cruz, sc-515081). HRP-bound secondary antibodies anti-mouse (Cytiva, NXA931V) and anti-rabbit (Cytiva, NA934V) were incubated for 1 h at room temperature, and the signals were developed using homemade ECL (0.1 M Tris–HCl pH8.5, 0.2 mM coumaric acid, 1.25 mM Luminol) and Fujifilm super RX (Fujifilm) films. Band intensities were quantified using ImageJ software.

### RNA extraction and quantitative real-time PCR (RT-qPCR)

RNA was extracted from cells using the Nucleospin RNA kit (Macherey–Nagel) or NZY Total RNA Isolation kit (NZYTech), following the manufacturer’s instructions. When working with mouse tissue, samples were snap-frozen in liquid nitrogen and transferred to lysis buffer from the RNA kit into homogenization tubes with ceramic beads. Tissue was homogenised using a FastPrep-24 5G Instrument (MP Biomedical) with two cycles of 30’’ at 6 m/s, following the manufacturer's instructions. The concentration was measured using a NanoDrop ND-1000 spectrophotometer. cDNA was obtained using Maxima H Minus cDNA Synthesis Master Mix (Thermo Fisher) with 500 ng of RNA and random primers. RT-qPCR reactions were carried out either using 2 × Taqman Fast Universal PCR Master Mix (Applied Biosystems) with specific TaqMan probes and UPL probes, or using 2 × NZY qPCR Green Master Mix (NZYtech, #MB125) with specific primers. QuantStudio™ 3 or QuantStudio™ 5 Real-Time PCR Systems (Applied Biosystems) were used to run the reaction. TaqMan probes Hs01096146_m1 and Hs02758991_g1 were used for human *METTL1* and *GAPDH* amplification, respectively, and Mm99999915_g1 was used for mouse *GAPDH* amplification. For TaqMan Master Mix reactions with UPL probes, the primers used were F-5’-GCTATGGTGGCTTGTTAGTGG-3’ and R-5’-CTTCACCCGAATCTCCAGAC-3’ for mouse *Mettl1*, and F-5’-AGCTGGGGCACCTGTCTAT and R-5’-GAGGATGAAGCGGTCATCAG for human *WDR4*. For SYBR green reactions, the primers used were F-5’-TCTTTGCCAGTACAGGAGCT-3’ and R-5’-GGGACACCTGGAATTCGTTG-3’ for human *ISG15*, F-5’-TGTTGCTGAGCCCTACAAGG-3’ and R-5’-GTCTGAATGGACTGCTCCCC-3’ for human *IRF9*, F-5’-CTTGTGCGTACTGTCCTTCG-3’ and R-5’-AGTGGGATGGTGGGTGTAAG-3’ for human ꞵ-catenin, F-5’-ACTGCTCTGTCGTCTCTACCT-3’ and R-5’-GGTAACAAGGGCCTAACCCT-3’ for human *KIF20A*, F-5’-ATGGCAGTCTGGCGGCTGAATT-3’ and R-5’-CCAAACCAGGCTGGCACAATTG-3’ for human *STAT1*, F-5’- TGTGAAAGTCTCAGTCTTGC-3’ and R-5’-AGTCTGATTGCGAAAACCTG-3’ for human *IFIT1,* F-5’- GCTGAGAATTGCACTGCAAC-3’ and R-5’-AGATAGGCCAGTAGGTTGCA-3’ for human *IFIT2,* F-5’-GTGGCTTTGCTCACTCATGT-3’ and R-5’-TGCTAACCACCGAGATGTCA-3’ for human *IFI44.* The ddCt values were measured and normalised against GAPDH values. Three technical replicates were used for this study. Biological replicates are indicated for each experiment.

### North-dot blotting

Total RNA was extracted from cells in culture or mouse prostate using TRIzol and isopropanol precipitation as previously described. tRNA size selection was performed using the mirVana isolation kit (Thermo Fisher) following the manufacturer’s instructions. 10 ng of small RNAs (< 200 nt) or large RNAs (> 200 nt) were blotted onto 0.2 μm nitrocellulose membranes (HE Healthcare) using Milliblot D (Millipore). Membranes were crosslinked with 120 mJ/cm^2^ in UV Stratalinker 2400 (Stratagene), then blocked with 5% BSA in RNA-free PBS-Tween for 1 h. Anti-m^7^G antibody (MBL, RN017M) was incubated overnight at 4 °C, followed by incubation with anti-mouse HRP-conjugated secondary antibodies at room temperature for 1 h. Signals were detected using homemade ECL. For loading control, membranes were stained with 0.04% methylene blue (Sigma) in 0.3 M Sodium acetate pH 5.2 for 10 min. The methylene blue staining intensity was used to normalize the X-ray films. To measure the m^7^G levels in mouse urine, fresh urine was collected and directly loaded onto nitrocellulose membranes, and the protocol was performed as previously described. Biological replicates are indicated for each experiment.

### Tissue immunohistochemistry and immunofluorescence

Tissues or tumours were collected and fixed overnight with 4% paraformaldehyde, transferred to 70% ethanol, embedded in paraffin and sectioned at a thickness of 5–10 μm. A standard immunohistochemistry or immunofluorescence protocol was employed and ImmPRESS™ reagents (Vector Laboratories) were used for immunohistochemistry. The slides were dewaxed and rehydrated with a battery of xylene and decreasing graded ethanol to water, and antigen retrieval was performed in a microwave in citrate buffer (pH 6) (Ki67, AR, K14, cleaved-Casp3, Arg1, iNOS, mouse CD68) or Tris–EDTA buffer (pH 9) (METTL1, pSTAT, mouse CD8, human CD8, CD163, and human CD86). Endogenous peroxidase activity was blocked by incubating the slides with 3% hydrogen peroxide (Emsure), this step was skipped in the immunofluorescence. PBS washes and permeabilization with 0,1% Triton was performed, followed by blocking with specific serum. Primary antibodies were prepared in blocking solution and incubated overnight at 4ºC with the following antibodies: anti-METTL1 (Invitrogen, PA5-54,280), anti-AR (Cell Signaling, 5153), anti-K14 (Covance, PRB-155P-100), anti-Ki67 (Vector Labs, VP-K451), anti-cleaved Casp3 (Cell Signaling, 9661S), anti-pSTAT1 (Santa Cruz, sc-8394), anti-CD68 (Biolegend, 137008), anti-mouse CD8 (Abcam, ab217344), anti-Arg1 (Cell Signaling, 93668), anti-iNOS (Abcam, ab15323), anti-CD68 (Santa Cruz Biotechnology, sc-20060), anti-CD163 (Leica, NCL-CD163), anti-CD86 (Cell Signaling, 91882), and anti-human CD8 (DAKO, IR623/IS623). For double immunofluorescence, one of the primary antibodies was incubated at 4 ºC overnight, followed by incubation with the second primary antibody at room temperature for 4,5 h. For immunohistochemistry, secondary antibody incubation was performed with ImmPress HRP kit (Vector Laboratories) and the reaction was visualised using ImmPact DAB Substrate or Red Substrate kits (Vector Laboratories). Finally, the slides were counterstained with hematoxylin and eosin (Millipore) and mounted with DPX (Sigma-Aldrich). For immunofluorescence, secondary antibody incubation was performed for one hour with anti-rabbit Alexa Fluor 488, anti-goat 488 or anti-rabbit Alexa Fluor 594 antibodies (Invitrogen). Nuclei were stained with DAPI (Sigma, D9542) and Vector® TrueVIEW® Autofluorescence Quenching Kit was used following the manufacturer's instructions for tissue autofluorescence reduction and slide mounting. For image acquisition, an Olympus BX-51 Microscope and Leica DM6 B Microscope were used for immunohistochemistry and immunofluorescence slides, respectively. To automate METTL1, CD8, CD86, and CD163 staining quantification in human PCa paraffin sections, 3,3'-diaminobenzidine (DAB) and haematoxylin channels were separated using the H-DAB option from Colour Deconvolution plugin of ImageJ software. DAB intensity was measured and optical density (OD) was calculated using the following formula: OD = log(max intensity/Mean intensity); where max intensity is 255 for 8-bit images. All other immune subtypes in mouse PCa paraffin sections were counted manually.

### mRNA expression profiling

For transcriptomic analysis of mouse prostate samples, RNA was extracted from the tissues using a Nucleospin RNA kit (Macherey–Nagel). Four biological replicates were used for genotype. *Pten-KO* mice were used at 3 and 6 months of age as well as aged-match wild-types. RNA quantity and quality were evaluated using a Qubit RNA HS Assay Kit (Thermo Fisher Scientific, Cat.# Q32855) and Agilent RNA 6000 Nano Chips (Agilent Technologies, Cat. # 5067–1511), respectively. Sequencing libraries were prepared using the “TruSeq Stranded Total RNA Human/Mouse/Rat” Kit (Illumina Inc., Cat. # RS-122-2201), followed by the “TruSeq Stranded Total RNA Sample Prep-guide (Part # 15031048 Rev. E)”. Briefly, starting from 500 ng of total RNA, rRNA was depleted and the remaining RNA was purified, fragmented, and primed for cDNA synthesis. The first strand cDNA was synthesised using SuperScript-II Reverse Transcriptase (Thermo Fisher Scientific, Cat. #18064-014). Second-strand cDNA was synthesised using Illumina reagents at 16 °C for 1 h. A-tailing and adaptor ligation were then performed. Finally, library enrichment was achieved using PCR. All the samples were multiplexed and sequenced on a HiSeq4000 platform (Illumina).

Sequenced reads were analysed using the nextpresso pipeline [140] as follows: sequencing quality was checked with FastQC v0.11.7 (http://www.bioinformatics.babraham.ac.uk/projects/fastqc/). Reads were aligned to the mouse reference genome GRCm38 with TopHat-2.0.10, using Bowtie 1.0.0 and SAMtools0.1.19, allowing two mismatches and twenty multihits. Read counts were obtained with HTSeq-count v0.6.1, using the mouse GRCm38 gene annotation. Differential expression was performed with DESeq2 using an FDR of 0.05. GSEAPreranked [141] was used to perform gene set enrichment analysis for several gene signatures on a pre-ranked gene list, setting 1000 gene set permutations. Only gene sets with significant enrichment levels (FDR q-value < 0.25) were considered.

For transcriptomic analysis of PC3 WT and *METTL1 KO* cells, total RNA was extracted from purified cell populations using the NZY Total RNA Isolation kit (RNA Purification, NZYTech) according to the manufacturer’s instructions. RNA integrity was assessed using an Agilent 2100 Bioanalyzer (Agilent Technologies, CA). Chip microarray hybridizations and data were generated with Affymetrix Clariom S Assay Human (Affymetrix) (> 20,000 well-annotated genes) were used in this study. Labelling and hybridization were performed according to Affymetrix protocols. Briefly, 100 ng of total RNA was amplified and labelled using the WT Plus reagent kit (Affymetrix) and then hybridised to the Clariom S human array (Affymetrix). Washing and scanning were performed using the GeneChip System of Affymetrix (GeneChip Hybridization Oven 645, GeneChip Fluidics Station 450, GeneChip Scanner 7G). Raw data were normalised by robust multiarray analysis (RMA) using the oligo package [142] and subsequently analysed to detect genes differentially expressed between PC3 *METTL1*-KO and WT cells through the limma package [143]. Differentially expressed genes in METTL1-KO vs. WT with Benjamini–Hochberg corrected *P* values < 0.05 were considered significant.

### PAR-CLIP

PAR-CLIP was adapted from the irCLIP protocol [132]. HA-METTL1-expressing PC3 cells were grown in 6-thioguanine (6SG) (Sigma-Aldrich) supplemented medium. Empty vector-infected cells were used as control. Cells were cross-linked at 365 nm (150 mJ/cm^2^) in Stratalinker 2400 and then lysed with CLIP Lysis buffer (50 mM Tris–HCl pH7.4, 100 mM NaCl, 1% Igepal CA-630, 0.1% SDS, 0.5% sodium deoxycholate in the presence of protease inhibitors) and passed 7–8 times through a 27G needle. For RNA digestion, two dilutions of RNase I (ThermoFisher Scientific, EN0602) were prepared in PBS (high: 1/50, and low: 1/250). 10μL of each dilution and 2μL of TurboDNase (ThermoFisher Scientific, AM2238) were added to 1 ml of each cell lysate (corresponding to ~ 1 mg of protein) and incubated for 3 min at 37 ºC, and then placed on ice for > 3 min. For HA-METLL1 immunoprecipitation 30 µl of Pierce anti-HA magnetic beads (ThermoFisher Scientific, #88836) were used. After washing, RNA 3’ ends were dephosphorylated using PNK (NEB, M0201L) and FastAP Alkaline phosphatase (ThermoFisher Scientific, EF0654). Next, the 3’ pre-adenylated adapters L3-IR-App were ligated with T4 RNA Ligase (NEB, M0437M). Adaptors were removed and RNAs were treated with RecJ_f_ endonuclease (NEB, M0264S). RNA–protein complexes were run on a NuPAGE 4–12% Bis–Tris gel (ThermoFisher Scientific, #NP0322BOX) and transferred onto a nitrocellulose membrane. RNA-bound complexes between 75 and 130 KDa were cut out from the membrane, and treated with proteinase K. After phenol extraction, RNAs were reverse-transcribed with Superscript IV (ThermoFisher Scientific, #18091050), and cDNA was circularized with CircLigase II (Biosearch Technologies, #CL9021K). PCR was performed with P3/P5 Solexa primers for 22 cycles, and the appropriate size of products were confirmed by gel electrophoresis. Sequencing of the libraries was performed on a HiSeq X instrument following standard manufacturer’s procedures, with single-read, 151-bases read profile.

### PAR-CLIP analysis

For the analysis of PAR-CLIP form PC3 cells, raw sequence data was demultiplexed based on library specific barcodes, quality filtered (mean sequence quality > 30) and trimmed (removing barcodes, PCR and sequencing adapters) using Flexbar (v.3.5.0) as described in [144].

The resulting reads were processed with the nf-core iCLIP 1.0.0 analysis pipeline (https://nf-co.re/clipseq). Briefly, after another round of trimming using Cutdapt (v3.0.0), reads are initially aligned with Bowtie2 (v2.4.2) against tRNA and rRNA sequences (https://github.com/nf-core/clipseq/blob/master/assets/small_rna/Homo_sapiens/Homo_sapiens.smallRNA.fa.gz). All unaligned reads are then mapped against the reference genome sequence (GRCh38, NCBI) using STAR (v2.6.1d). Potential PCR duplicates are removed based on their position and UMI using UMI-tools (v1.1.1). Reads were quantified against tRNA and rRNA sequences and genome annotation GRCh38 (NCBI) by simple overlap using bedTools (v2.3). The counts were subsequently normalized by library size (RPM) and transcript length (RPKM). We counted uniquely and commonly detected genes between both conditions (RPKM > 0) and estimated fold-change enrichment of reads for the latter. All statistical analysis and visualizations were performed using base R and the R package ggplot2.

PAR-CLIP data from the HEK293T cell line expressing Flag-METTL1 were obtained from the GSE100756 [51]. The data were downloaded from the UCSC table browser. Reads were aligned to the hg19 genome assembly using bowtie version 1.2.3 with the configuration recommended by PARalyzer developers [145], -v 2 -m 10 –best –strat. The reads were aligned to Repeat Masker sequences after removing the tRNA sequences. Aligned reads were used for peak calling with PARalyzer version 1.5 using the recommended settings. The peaks identified in only one sample were removed. Counts per million associated with the common peaks were used to calculate the coefficient of variation (CV). Only the peaks with a CV lower than 30% were retained. These peaks were annotated according to GENCODE release 19 for further analysis using in-house scripts.

### AlkAniline-seq

For m^7^G detection at single-nucleotide resolution, AlkAniline-seq was performed according to [52]. Briefly, RNA was prepared from two independent replicates of PC3 WT and PC3 *METTL1 KO* cells. tRNA fraction (150 ng) was subjected to alkaline hydrolysis in 50 mM bicarbonate buffer pH 9.2 for 5 min at 96 °C. The reaction was stopped by ethanol precipitation using 3 M Na-OAc, pH 5.2 and glycoblue as a carrier in liquid nitrogen. After centrifugation, the pellet was washed with 80% ethanol and resuspended in nuclease-free water. RNA fragments were dephosphorylated using 5 U of Antarctic Phosphatase (NEB, #M0289S) for 1 h at 37 °C. After inactivation of the phosphatase for 5 min at 70 °C, RNA was recovered by a phenol:chloroform:isoamyl alcohol (25:24:1) extraction, followed by a chloroform step and precipitated in 96% ethanol using 3 M Na-OAc, pH 5.2 and glycoblue for at least 2 h at -80 °C. After centrifugation, the pellet was washed with 80% ethanol and resuspended in 20 μL of Aniline 1 M pH 4.5 and incubated in dark for 15 min at 60 °C. The reaction was stopped by ethanol precipitation and incubated overnight at -80 °C. After centrifugation, the pellet was washed twice with 80% ethanol and resuspended in nuclease-free water. RNA fragments were converted to library using NEBNext® Small RNA Library kit (NEB, #E7330S) following the manufacturer's instructions. DNA library quality was assessed using a High Sensitivity DNA chip on a Bioanalyzer 2100. Library quantification was done using a fluorometer (Qubit 2.0 fluorometer, Invitrogen, USA). Libraries were multiplexed and subjected for high-throughput sequencing using an Illumina Nextseq2000 instrument with a 50 bp single-end read mode.

Bioinformatic analysis of AlkAnilineSeq data was performed as follows. Raw sequencing reads were trimmed by Trimmomatic v0.39, to remove eventual primer dimers and 3’-adapter sequence. Trimmed reads were aligned by bowtie2 v2.4.2 in end-to-end mode to the optimized human tRNA reference containing 43 representative tRNA species [146] (and positions of reads’ 5’-extremities were counted using a custom awk script. Normalized cleavage (NCleavage) was calculated for every RNA species, by dividing of reads’ count for a given position to total number of reads mapped to a given RNA [147].

### tRNA-seq

For tRNA-seq, tRNA libraries were prepared from PC3 WT and *METTL1 KO* cells with four independent biological replicates per condition. Total RNA was extracted using TRIzol (Honeywell, 33539), followed by DNAse I Turbo (Invitrogen) treatment. Then, tRNAs (< 200 bp) were selected using the mirVana miRNA isolation kit (Thermo Fisher) following the manufacturer’s instructions. tRNAs were de-aminoacylated with a solution containing 1 mM EDTA and 0.1 M Tris–HCl pH 9.0 for 30 min at 37 °C. All small RNAs were treated with T4 PNK (NEB) to ensure the presence of phosphorylated 5’ ends and 3’OH ends. tRNA libraries were generated using the NEXTFLEX Small RNA-Seq Kit v3 (Bioo Scientific Corp.) for Illumina Platforms, following the manufacturer instructions. All the samples were multiplexed and sequenced on a HiSeq2500 platform (Illumina).

### tRNA-seq analysis

Raw sequencing FASTQ files were trimmed with ‘*cutadapt’* of the ‘TGGAATTCTCGGGTGCCAAGG’ adapter, retaining reads with a minimum length of 23 nt. Reads were subsequently trimmed by 4 nt on both ends following the platform recommendations. Reads were aligned to the GRCh38 (hg38) reference genome using *bowtie* (bowtie-bio.sourceforge.net) with parameters ‘-m 500 -v 2’, allowing a maximum of 500 multiple mappings and two mismatches. tRNA genomic coordinates for hg38 were obtained from the ‘*GtRNAdb’* database (gtrnadb.ucsc.edu). Reads on tRNA coordinates were processed using ‘*htseq-count’* from the Python package ‘*HTSeq*’ (htseq.readthedocs.io) with the *‘-samout’* option, and counts for individual reads representing tRNA fragments and their multiple sequence alignments on tRNAs were extracted from the resulting sam files using custom PERL scripts. Fragments with fewer than 10 counts over all samples were removed, and a pseudo-count of 1 was added. Fragments were filtered according to length: ≥ 18 nt and ≤ 50 nt. Counts of tRNA fragments were normalized, and differential abundance of tRNA fragments was evaluated in 3 replicates per condition (WT or KO) using the R package ‘*DESeq2’* (https://bioconductor.org/packages/release/bioc/html/DESeq2.html). 3' tRNA-derived fragments (3’-tRFS) were defined as tRFs that end < 10 from tRNA end site, Int-tRFs span the interior of the mature tRNA sequence, 5′tRNA-derived fragments start site > -10 and < 10 of the tRNA start site, from which 5’-halves were > 35 nucleotides long, and all others were < 35 nucleotides and were split into 5’-tRFs (without 5′ terminal oligoguanines) and 5’TOGs (containing a 5′ terminal oligoguanines).

### Northern blotting

Total RNA was extracted from cultured cells using TRIzol and isopropanol precipitation, as previously described. To analyse tRNA fragment formation under stress conditions, 200 μM NaAsO_2_ was added to the cells for 2 h, then NaAsO_2_ was removed and the cells were collected at the indicated time points (h) after NaAsO_2_ was added to cells. The 15% polyacrylamide 8 M urea-TBE gels were pre-run for at least 1 h at 180 V in 1 × TBE. 8 to 10 μg of total RNA were loaded onto 15% polyacrylamide gels, run for 1 h at 150 V, and transferred to a Nylon Hybond-NX membrane (GE Healthcare) for 1 h at 45 V by wet electrophoresis (BioRad) in 0.5 × TBE. Membranes were crosslinked with 120 mJ/cm^2^ in UV Stratalinker 2400. Membranes were pre-hybridised at 42ºC for at least 1 h in ULTRAhybond buffer (Invitrogen). Single-stranded DNA probes (20 pmol) were radiolabelled at the 5’ end by incubating 80 mCi ^32^P-ATP (Perkin Elmer) with T4 PNK (NEB) for 1 h at 37ºC. Probes were purified using Oligo Clean and Concentrator (Zymo Research) columns and denatured for 5 min at 95ºC. Membranes were incubated overnight with the radiolabelled probe and then washed twice with low-stringent buffer (0.1 × SSC, 0.1% SDS) at 42 ºC for 15 min and twice with high-stringent buffer (2 × SSC, 0.1% SDS). Membranes were dried and exposed overnight at -80ºC with X-ray films. The following DNA probes were used: 5’-Cys-TACCCCTGAGCTATACCCCC-3’, 5’-Pro-ATACCCCTAGACCAACGAGCC-3’, 5’-Lys- CCGACTGAGCTAGCCGGGC-3’. Two biological replicates per cell line were analysed.

### Flow cytometry

To measure protein synthesis, cultured cells were incubated with 20 μM OP-puro (Medchem Source LLP) for 1 h at 37 ºC in complete medium. Cells were untreated or treated with 200 µM NaAsO_2_ for two hours, then NaAsO_2_ was removed and cells were collected after 2, 4 or 8 h after NaAsO_2_ was added to cells. Cells were collected by trypsinisation and fixed with 1% paraformaldehyde in PBS for 15 min on ice. The cells were washed in PBS and permeabilised with PBS supplemented with 3% fetal bovine serum (Sigma-Aldrich) and 0.1% saponin (Santa Cruz Biotechnology) for 5 min at room temperature. OP-puro conjugation to a fluorochrome was performed by azide-alkyne cycloaddition using the Click-iT Cell Reaction Buffer Kit (Jena Bioscience) and 5 μM of Alexa Cy5.5 conjugated to azide (Jena Bioscience). After 30-min reaction, cells were washed twice in PBS with 3% FBS and 0.1% saponin and then resuspended in PBS. Events were acquired using an Accuri C6 cytometer. Staining and protein synthesis were analysed using FlowJo software (BD Biosciences).

For cell cycle analysis, cells in culture were trypsinised, washed with PBS and medium, fixed in cold 70% ethanol for 10 min, pelleted and resuspended in staining buffer containing 1 µg/mL Propidium Iodide and 50 µg/mL RNase A (Roche) in PBS. The cells were then incubated for 30 min at 4 ºC in the dark and acquired using a BD Accuri C6 flow cytometer (BD Biosciences). Cell debris and aggregates were eliminated by gating the cells forward versus side scatter and FSC-H versus FSC-A. The DNA content was quantified using FlowJo software (BD Biosciences). Three technical replicates were used for each cell type.

For cell death and apoptosis analysis, 70.000 cells were seeded in 12-well plates, after 48 h cells were washed twice with PBS and trypsinised. Cells were pelleted and resuspended in Annexin V Binding Buffer (10 nM Hepes/NaOH (pH 7,4), 140 mM NaCl 2,5 mM CaCl2) containing 1 mg/mL Propidium Iodide and 10 mg/mL RNase A (Roche). 3 uL of Annexin V (Immunostep, ANXVF-200 T) was added to each sample. Cells were then incubated for 15 min at room temperature in the dark and after that 50 uL of Annexin Binding Buffer was added to each sample. The cells were acquired using a BD Accuri C6 flow cytometer (BD Biosciences). Cell debris and aggregates were eliminated by gating the cells forward versus side scatter and FSC-H versus FSC-A respectively. The percentage of apoptotic cells was quantified using FlowJo software (BD Biosciences). Three technical replicates were used for cell type.

### Cap binding assay

m^7^G-cap pulldown was performed as described [57]. Briefly, subconfluent WT PC3 cells were collected in lysis buffer (10 mM Tris–HCl, 140 mM KCl, 4 mM MgCl_2_, 1 mM DTT, 1 mM EDTA, 1% NP-40, protease inhibitors and homemade phosphatase inhibitors) and incubated on ice for 30 min. Lysates were cleared by centrifugation at 12,000 rpm for 30 min at 4 ºC and quantified using BCA method (Thermo Fisher Scientific). 30 μL of 7-methyl-GTP-Sepharose beads (Jena Bioscience) were washed and incubated with 200 μg of protein extracts diluted in lysis buffer to 1 mg/mL. Incubation was performed overnight with rotation at 4 ºC. The beads were then washed twice with lysis buffer containing 0.5% NP-40 and twice with PBS. Bound proteins were analysed by western blotting.

### Transfection of synthetic 5'TOG, anti-TOG RNA oligonucleotides and RNAi

Cells were seeded in 6-well or 10 cm plates and transfected with INTERFERin (Polyplus) following the manufacturer’s instructions. 24 or 48 h before collection, the cells were transfected with 25 nM of either scramble control, 5′TOG, or an anti-TOG oligonucleotide. The RNA oligonucleotides (from IDT) used for the transfection were TOG-5Cys: 5’-GGGGGUAUAGCUCAGGGGUGAGCAUUUGACUG-3’, Anti-TOG-5Cys: 5’-CAGUCAAAUGCUCUACCCCUGAGCUAUACCCCC-3’, or AllStars Negative Control siRNA (Qiagen, 1027280) as controls. RNAi against human METTL1 used were Hs_METTL1_3 FlexiTube siRNA (Qiagen, SI00076132). RNAi against human STAT1 used were STAT1-siRNA.7 (Qiagen, SI02662884).

### Biotinylated 5'TOG oligonucleotide pulldown

Two 10 cm tissue culture plates of WT PC3 cells per condition were transfected with 40 nM of a mix 1:1 of scramble + 5′TOG oligonucleotide, or 5′TOG + Anti-TOG nucleotide using INTERFERin (Polyplus) reagent. Twelve hours after transfection, cells were washed twice with ice-cold PBS, UV-crosslinked at 254 nm with 150 mJ/cm^2^ and harvested on ice. The cells were centrifuged and resuspended in lysis buffer (50 mM Tris–HCl pH 7.4, 100 mM NaCl, 0.5% Triton X-100, 0.5% sodium deoxycholate, 0.1% SDS, and 5 mM EDTA, supplemented with 0.1 U/μL RNase inhibitors and protease inhibitors). Lysates were sonicated three times for 10 s at 10 W and then cleared by centrifugation at 12,000 rpm for 12 min. 600 μg of extracts were incubated with pre-washed streptavidin-C1 Dynabeads (Thermo Fisher Scientific) for 2 h in rotation at 4 ºC. After incubation, beads were washed once with lysis buffer, once with high-salt buffer (50 mM Tris/HCl, 1000 mM NaCl, 0.5% Triton, 0.25% sodium deoxycholate, 1 M Urea, 5 mM EDTA, 1 mM DTT) and once with PNK/Tween buffer (50 mM Tris, 10 mM MgCl_2_, 0.2% Tween-20). The beads were then centrifuged and resuspended in 1.5 × Laemli buffer supplemented with 100 mM DTT, and eluted by boiling at 75ºC for 10 min with shaking at 1100 rpm. Proteins were loaded onto a 10% SDS-PAGE gel and subjected to western blotting.

### Proliferation assays

From 3.000 to 15.000 cells were seeded in 12-well plates, with three or six replicates per condition. Cells were maintained in culture medium for up to 8 days, with collection points every two days. Day 0 was defined less than 24 h after seeding. In the case of the catalytic dead mutant proliferation rescue, 0.1 µg/mL of doxycycline was added to the cells during curve seeding. For collection, cells were washed twice with PBS and fixed with 4% paraformaldehyde (Panreac). Staining was performed with 0.1% crystal violet (Sigma) in 10% methanol for 1 h, after which the wells were thoroughly washed with dH_2_O and dried overnight at room temperature. Crystal violet was dissolved in 10% acetic acid for 40 min, and cell density was quantified by measuring the absorbance at 595 nm using a Plate Infinite reader 200 Pro (TECAN). All data were normalised to day 0 measurements.

### Single-cell spheroids generation

To prevent cell adhesion, p96-well plates were treated with 12 g/L poly-2-hydroxyethyl methacrylate (polyHEMA, Santa Cruz Biotechnology) resuspended in ethanol. Once the plates were dry, the cells were seeded at density of 1 cell per well. Spheroids were growth for 18–21 days in DMEM/F12 (Gibco) medium supplemented with 1% B27 (Gibco), 0.02 µg/mL EGF (Gibco), 0.004 µg/mL bFGF (ThermoFisher), 8% BSA (Nzytech), and 1% penicillin/streptomycin. Images were taken using a Nickon Eclipse Ti-S microscope and a JenoPTIK ProgRes C3 camera. Spheroid volume was estimated using the formula: volume = (length x width)^2^ × 0.526.

### Cell immunofluorescence of BrdU

Cells were seeded on cover glasses (Thermo Scientific) and grown in complete medium. For BrdU incorporation in S phase, the cells were treated with 10 μM BrdU (Sigma, 19–160) for 1 h. For cell collection, PBS washes were performed, followed by fixation for 10 min with 4% PFA (Panreac Applichem) and permeabilization with 0.2% Triton X-100/PBS (Panreac Applichem) for 10 min. For BrdU staining, coverslips were treated with 2N HCl for 30 min at room temperature and then washed thoroughly. Blocking was performed for 1 h at room temperature with 5% goat serum (Gibco) in PBS. Primary antibodies diluted in blocking solution were incubated overnight at 4 ºC, using the following primary antibodies: anti-BrdU (Abcam, ab6326). The next day, the cover glasses were washed three times with 0.05% Tween-20 in PBS and incubated for 1 h with fluorophore-conjugated secondary antibodies at room temperature in the dark. Donkey/goat anti-mouse or anti-rabbit secondary antibodies conjugated to Alexa Fluor 488 or Alexa Fluor 594 were used. Nuclei were stained with 0.5 μg/mL of DAPI (Sigma, D9542) for 10 min, and cover glasses were mounted with Mowiol (Sigma, 81381). Fluorescence images were acquired with a Leica SP5 confocal microscope.

### Proteomic analysis

For total protein analysis, a sub-confluent p100 plate was collected per condition. Cells were trypsinised and washed three times with PBS, and cell pellets were snap-frozen in liquid nitrogen. The cells were then lysed in buffer containing 2 M thiourea, 7 M Urea, 4% Chaps and 200 mM DTT. The extracts were cleared by centrifugation**.** For actively translated proteins, 24 × 10^6^ cells were treated with 20 μM OP-puro (Medchem Source LLP) for 1 h at 37 ºC in complete medium and snap-frozen in liquid nitrogen after collection. The alkyne-tagged proteins were captured in an azide-tagged resin by a Cu(I)-catalysed azide-alkyne cycloaddition reaction, which was performed using a Click Chemistry Capture Kit (Jena Bioscience). Bound proteins were then washed with high-stringency buffer and detached from the resin upon protease digestion. Mass spectrometry was performed to analyse protein composition.

For in-solution digestion, the proteins were extracted using 7 M urea, 2 M thiourea, and 4% CHAPS. Samples were incubated for 30 min at RT under agitation and digested according to the FASP protocol described previously [148] with minor modifications. Trypsin was added at a trypsin:protein ratio of 1:10, and the mixture was incubated overnight at 37 °C. For on-bead, the proteins were denatured in 8 M urea, reduced with DTT (5 mM), and alkylated with iodoacetamide (25 mM). The sample was diluted in ammonium bicarbonate to 1.5 M urea, and trypsin was added to the same ratio described above and incubated overnight at 37 °C as described elsewhere. The resulting peptides in either strategy were concentrated in a speed-vac and desalted using C18 stage tips (Millipore) prior to acquisition. For mass spectrometry analysis, the samples were analysed using a hybrid trapped ion mobility spectrometry – quadrupole time of flight mass spectrometer (timsTOF Pro with PASEF, Bruker Daltonics) coupled online either to a nano Elute (Bruker) or EvoSep ONE liquid chromatograph (EvoSep). Protein identification and quantification were carried out using MaxQuant software [149] with default settings, except for LFQ min ratio count of 1. Searches were performed against a database consisting of human protein entries (Uniprot/Swissprot) with precursor and fragment tolerances of 20 ppm and 0.05 Da respectively. Only proteins identified with at least two peptides with an FDR < 1% were considered for further analysis. Data (LFQ intensities) were loaded onto Perseus platform [150] and further processed (log_2_ transformation and imputation).

The Gene Ontology web tool powered by Panther (http://geneontology.org/) was used for functional annotation analysis of differentially expressed gene sets to identify specific gene subsets sharing co-occurrent functional annotations linking them, with high statistical significance, to particular Gene Ontology (GO) Biological Process categories. Fisher's exact test was applied with Bonferroni correction.

### Polysome profiling

Six 150 cm plates were seeded per replicate, and 80% confluent plates were treated for 5 min at 37 °C with 0.1 mg/mL of cycloheximide (CHX, Santa Cruz Biotechnology). Plates were washed twice with PBS supplemented with 0.1 mg/mL CHX and then collected with lysis buffer (20 mM Tris–HCl, 100 mM KCl, 5 mM MgCl_2_, 0.3% Igepal, 0.1 mg/mL CHX, protease inhibitors). RNA–protein content was measured using a Smart-Spect Plus spectrophotometer (Bio-Rad) and equal amounts of extracts were loaded into the gradients. 7–50% sucrose gradients (20 mM Tris–HCl pH7.5, 100 mM KCl, 5 mM MgCl_2_, 1% CHX) were previously prepared and balanced, and translation components were separated by ultracentrifugation for 2:45 h at 4 ºC and 39,000 rpm with no break. Gradients were fractionated using a Density Gradient Fractionation system (Brandel) and 500 μL fractions were collected. Half the volume of each fraction was used for RNA extraction with warm phenol protocol. Briefly, equal volumes of the fractions were mixed with 60 ºC-heated Phenol–Chloroform and a solution containing 10 mM Tris, 350 mM NaCl, 10 mM EDTA, 1% SDS and 42% urea (Fisher Chemical), followed by RNA precipitation with isopropanol. cDNA and RT-qPCR were performed on 16 different fractions from each replicate. To calculate the content of mRNA in each separated fraction, the mRNA content of each transcript in each fraction was normalized with the content of the transcript in all the analysed fractions and represented as the proportion or fraction of the total.

### Cytokine array

For human cells, on day 0, PC3 WT and *METTL1 KO* cells were seeded in 6-well plates. On day 3, the medium was removed and 1 ml of serum-free DMEM was added to each well. On day 5, the medium was collected and centrifuged again. The supernatant was used to analyse the expression levels of multiple proteins using the RayBio C-series Human Inflammation Antibody Array 3 Kit (RayBiotech, AAH-INF-3–2). For mouse tissues, prostate tissue from five-months old *Pten-KO/Mettl1* + */* + and *Pten-KO/Mettl1*^*fl/fl*^ mice were used. Protein extracts from three mice of each genotype were used to analyse multiple protein expression levels using Mouse Cytokine Array G3 kit (RayBiotech, AAM-CYT-G3-8).

### M1/M2 macrophage polarisation of THP1 cells

50.000 THP-1 cells were seeded in 6-well plates at day 0, adding 25 nM phorbol 12-myristate 13-acetate (PMA) added to differentiate monocytes to Mø macrophages. At day 1, PMA were removed, at day 2 conditioned media from PC3 WT and *METTL1 KO* cells was added. On day 5, cells were detached with TripLE express (Gibco) and polarisation was analysed using a BD FACSAria III flow cytometer (BD Biosciences). As controls, some cells were polarised to M1 by adding 100 ng/mL LPS and 20 ng/mL IFNγ, and another to M2 by adding 20 ng/mL IL-4 and 20 ng/mL IL-13. Antibodies against anti-CD11b (BioLegend, 101206), anti-CD14 (BD-Horizon, 562335), anti-CD68 (BioLegend, 333814), anti-CD86 (BioLegend, 374208), and anti-CD206 (BioLegend, 141708) were used. Cell debris and aggregates were eliminated by gating the cells forward versus side scatter and FSC-H versus FSC-A respectively. Polarisation was analysed using FlowJo software (BD Bioscience). Three technical replicates were used for cell type.

### Phagocytosis assay

The cells were treated in the same manner as for the polarisation assay, but this time 10.000 THP-1 were seeded in 24-well plates. On day 5, the medium was replaced with HBSS medium supplemented with 2% FBS and 50 ug of pHrodo™ Red E. coli BioParticles™ Conjugate for Phagocytosis (Thermo Fisher) was added to each well. Fluorescence intensity was measured for 4 h taking pictures every 5 min with an Eclipse TE2000 Inverted Microscope (Nikon).

### Monocyte isolation from peripheral blood and macrophage polarisation

PBMC were isolated from blood samples by centrifugation using the density gradient medium Lymphoprep™ (Stem Cell Technologies). Monocytes were isolated from PBMC using anti-human DynabeadsTM CD14+ (Thermo Fisher), according to the manufacturer’s instructions. 500.000 monocytes were seeded in 6-well plates at day 0, adding 5 ng/mL Granulocyte–Macrophage Colony-Stimulating Factor (GM-CSF) was added in order to differentiate monocytes to Mø. On day 3, conditioned media from PC3 WT and *METTL1 KO* cells was added. As controls, on day 6, some cells were polarised to M1 by adding 100 ng/mL LPS and 20 ng/mL IFNγ, and others to M2 by adding 20 ng/mL IL-4 and 20 ng/mL IL-13; M2 controls were seeded on day 0 with 50 ng/ml (Macrophage Colony-Stimulating Factor (M-CSF) instead of GM-CSF. Some cells were cultured for 6 days with medium and 5 ng/mL GM-CSF as the Mø control. On day 7, cells were detached with PBS containing 2.5% FBS (Gibco) and 0.5 Mm EDTA for 30 min, and polarisation was analysed using a BD FACSAria III flow cytometer (BD Biosciences). Antibodies against anti-CD11b (BioLegend, 101206), anti-CD14 (BD-Horizon, 562335), and anti-CD68 (BioLegend, 333813) were used. Cell debris and aggregates were eliminated by gating the cells forward versus side scatter and FSC-H versus FSC-A respectively. Polarisation was analysed using FlowJo software (BD Bioscience). Three technical replicates were used for condition.

### T cell isolation from peripheral blood, proliferation and migration assays

PBMC were isolated from blood samples by centrifugation using the density gradient medium Lymphoprep™ (StemCell Technologies). PBMC cells were cultured on day 0 in a 75 mm^3^ flask adding 100U/mL IL-2 (Miltenyi Biotec) every 2 days. On day 7, cells were collected and stained with the Cell Trace CFSE Cell Proliferation Kit (Thermo Fisher), as indicated by the manufacturer.

For proliferation measurements, lymphocytes were then co-cultured with 500.000 cells/well to previously polarised macrophages following the macrophage polarisation protocol described above. On day 9, the supernatant containing the lymphocytes was collected and proliferation was analysed using a BD FACSAria III flow cytometer (BD Biosciences). Antibody anti-CD3 (BioLegend, 300428) was used to select T lymphocytes.

For migration assays, peripheral blood monocytes were seeded in 24-well plates and polarised with PC3 WT and *METTL1 KO* cells conditioned or with specific cytokines as previously described. On day four of seeding, TC-inserts with a pore size of 8 μm (Sarstedt, 83.3932.800) were placed in each well. The same number of lymphocytes previously stained with CFSE was seeded in the upper compartment. After three hours, the medium from the lower compartment was collected, the volume was analysed using a BD FACSAria III flow cytometer (BD Biosciences), and the number of CFSE-positive cells was used as an indicator of lymphocyte migration. Monocytes and lymphocytes were isolated from the same healthy donors in each experiment.

### Analysis of abundance of cell types in human tumours

CIBERSORTx [151] and TIMER2.0 [89] were used to assess cellular abundance and cell type-specific gene expression patterns from bulk tissue transcriptome data obtained from The Cancer Genome Atlas Prostate Adenocarcinoma (TCGA-PRAD) collection [152] using cBioPortal [153, 154] and Taylor datasets [6]. For correlation analyses, only tumours with high *METTL1* expression were analysed. For TCGA samples, *METTL1* > 500 normalised RPMs. For Taylor samples, *METTL1* > 6.85 log_2_ normalised expression.

### Anti-PD1/CTLA-4 and anti-CD8 mouse treatments

At the age of four months, a combination of anti-mouse PD1 (Bio X Cell, BE0146) and anti-mouse CTLA-4 (Bio X Cell, BE0131) was administered at a dose of 5 μg of each antibody per gram of mouse, both antibodies in 100 μL. To eliminate CD8 T cells, anti-mouse CD8α (Bio X Cell, BE0061) was administered at a dose of 100 μg per mouse. As control treatment, a combination of rat IgG2a isotype control (Bio X Cell, BE0089) and polyclonal Syrian hamster IgG (Bio X Cell, BE0087) was used as a control at the same dose. All treatments were administered by intraperitoneal injection every three days for eighteen days. Mice were weighed every three days to discard treatment toxicity. The in vivo response to immune checkpoint blockade or CD8 T cells inhibition was evaluated by measuring each prostate lobe volume one week after the treatment regime was completed.

### Statistical analysis

Statistical analyses were performed using GraphPad Prism 8.2. *P* values less than 0.05 were considered statistically significant. For in vitro experiments, continuous and normal distribution was considered; applying unpaired one (validation or hypothesis-driven experiment) or two (experimental design without predicted result) tailed Student’s t-test, whenever indicated. In vivo experiments with ten or less replicates were considered normally distributed, and a parametric Student’s t-test was applied. For more than ten replicates, unpaired Student’s t-test was applied if the distribution was normal and Mann–Whitney test if the distribution was not normal. For the comparison of multiple samples, a one-way or two-way ANOVA test was applied. Data are represented as mean ± s.d. or s.e.m. when indicated, mean with interquartile range or boxplot with line at mean or min and max. Pearson’s correlation was computed for normally distributed samples and Spearman’s correlation for non-normally distributed samples. “*n”* represents the number of independent experiments, individual mice or patient specimens. At least three technical replicates were used. No statistical analysis was performed to determine the sample size. The experiments were randomized. The investigators were not blinded to allocation during the experiments and outcome assessment.

### Data access

The transcriptomic data, PAR-CLIP and microarray generated in this publication have been deposited in NCBI’s Gene Expression Omnibus (GEO Series accession numbers GSE189883, GSE190911, and GSE230694) and the European Nucleotide Archive (ENA projects PRJEB49285 and PRJEB53441). The mass spectrometry proteomics data were deposited in the ProteomeXchange Consortium via the PRIDE partner repository with the dataset identifier PXD030141.

### Supplementary Information

**Additional file 1: Supplementary Figure S1.** Expression of RMPs in prostate tumours. **A)** mRNA expression of RMPs is significantly altered in human normal prostate tissue (N), primary (PT), and metastatic tumour (M). Y-axis shows log_2_-normalised expression data from Grasso et al*.* [7],Taylor et al*.* [6], and Varambally et al*.* [42] datasets (left panels). Kaplan–Meier curves showing disease-free survival (DFS) of patient groups selected according to decile expression of the indicated RMP genes (middle panel). The right panel shows differential expression in disease-free (DF) and recurrent patient samples from TCGA dataset [3]. **B, C)** Kaplan–Meier curves representing the disease-free survival (DFS) of patient groups selected according to the decile expression, data from the TCGA dataset [6] (TCGA: *n* = 491). **D, E)** PCa cell lines show increased METTL1 protein and mRNA levels. Western blotting showing METTL1, WDR4, AR, PTEN and phosphor-S6K expression (**D**), and qPCR (**E**) of normalised mRNA expression (lower panel) in prostate epithelial cell lines (PWR-1E and RWPE-1), benign prostatic hyperplasia cell lines (BPH1), and prostate cancer cell lines (VCap, C4-2, LNCaP, 22Rv1, DU145, and PC3). Mean ± SD (**D, E**). Statistical tests: ANOVA test (**A**), log-rank Cox test (**A, B**); Student’s t-test with Welch’s correction, grouping all data from benign vs. all data from tumour cells ****p < 0.0001 (**E**).

**Additional file 2: Supplementary Figure S2.** Regulation of *METTL1* expression in PCa. **A)** Correlation analysis between *METTL1* and *KLK3* expression, and *METTL1* and *AR* expression in the human primary PCa expression datasets. The plotted values correspond to the log_2_-normalised expression values. Black line represents linear regression, grey area indicates the limits of the confidence intervals. Pearson's correlation coefficient (R) and *p*-values are indicated. Grasso *n* = 88; Taylor *n* = 183; TCGA *n* = 497. **B)** No effect on mRNA expression levels of *METTL1* upon dihydrotestosterone (DHT) treatment in LNCaP cells. Mean ± SD, *n* = 3. **C)** Graphical representation of PI3K inhibition by different compounds. Small molecule inhibitors used: pan-PI3K inhibitor BKM-120 (BKM), AKT inhibitor MK2206 (MK), mTORC1 inhibitor rapamycin (RAPA), and mTORC1/2 inhibitor Torin (TOR). **D, E)** *METTL1* expression is affected by the inhibition of downstream PI3K pathway members. Western blotting (**D**) and RT-qPCR (**E**) analyses of *METTL1* expression upon PI3K pathway inhibition in PC3 cells. The cells were treated for 48 h (**D**) or 8 h (**E**). Mean ± SD, *n* = 2 (**D**) and *n* = 6 (**E**). **F)** Representative images of immune stained sections for Mettl1 and markers for luminal (AR), and basal (K14) cells in *Pten-KO* prostates (dorsal and ventral lobes) in invasive carcinoma (6 months old mice) and in aged-match wild-type (WT) prostates*.* Scale bars represent 50 μm. Statistical tests: One-tailed Student’s t-test (**B, D, E**). *p < 0.05, **p < 0.01, ***p < 0.001.

**Additional file 3: Supplementary Figure S3.** tRNAs are methylated at guanine-7 by METTL1. **A)** Western blot showing the expression of doxycycline-inducible HA-METTL1 in PC3 cells. **B, C)** Density distribution of reads per million (RPM) identified after PAR-CLIP analysis of METTL1-bound tRNAs (**B**) and other RNA species (**C**) in PC3 (control) and HA-METTL1 expressing PC3 cells. The red line indicates the third lower quartile of total RPMs. **D)** tRNAs are the most common RNA species that bind Flag-METTL1 in HEK293T cells. Density distribution of reads per million (RPM) identified after PAR-CLIP analysis of METTL1-bound RNAs: The red line indicates the third lower quartile of total RPMs. Data were retrieved from Bao et al*.* study. **E)** tRNAs comprise half of the reads of all RNAs bound to METTL1 after PAR-CLIP analysis in HEK293 cells. **F)** Boxplot representing the median with interquartile range of reads per million (RPM) per transcript bound to METTL1 in HEK293T cells. **G)** Schematic representation of genomic editing introduced by CRISPR-Cas9 technology in the PC3 *METTL1 KO* clones used in this study. **H)** *METTL1* mRNA expression levels in *METTL1 KO,* and WT control clones used in this study. Mean ± SD, *n* = 3. **I)** Graphics representing the experimental workflow followed to generate AlkAniline-seq libraries. **J)** Normalised cleavage signals for METTL1-methylated tRNAs Cys and Ile, and METTL1 non-methylated tRNAs His and Glu in PC3 WT and *METTL1 KO* cells.

**Additional file 4: Supplementary Figure S4.** Accumulation of 5'tRNA fragments in *METTL1 KO* cells. **A)** The most common sequences of 5' terminal guanines (5G) containing 5'TOGs and 4G 5'TOGs are shown. **B, C)** Northern blot showing the absence of 5'tRNA fragments derived from Lys and Pro tRNA in PC3 WT *METTL1 KO* cells under normal conditions. **D)** qPCR showing reduced expression of *METTL1* in 22Rv1 cells upon *METTL1* silencing using siRNA. Mean ± SD, *n* = 3. **E)** Dot blot against m^7^G showing reduced methylation levels in tRNAs extracted from *METTL1*-silenced 22Rv1 cells. Mean ± SD, *n* = 3. **F)** Northern blot detection of Cys-derived 5'TOGs in *METTL1*-silenced 22Rv1 cells. Mean ± SD, *n* = 3. **G)** Northern blot detection of Cys-derived 5'TOGs in PC3 *METTL1 KO* cells (second biological replicate), unexposed (0 h) or after 2 and 8 h of oxidative stress exposure. **H)** Northern blot detection of Lys-derived 5'TOGs in PC3 *METTL1 KO* cells unexposed (0 h) or after 2 and 8 h of oxidative stress exposure. **I)** Western blotting of DU145 *METTL1 KO* and WT cell clones generated using CRISPR-Cas9 technology and parental DU145 (DU) cells. **J)** Northern blot detection of Cys-derived 5'TOGs in DU145 *METTL1 KO* cells (second biological replicate), unexposed (0 h) or after 2 and 8 h of oxidative stress exposure. The loading control by red-safe staining (tRNA) is shown in the bottom panels (**B, C, F–H, J**). Bands corresponding to full-length tRNAs are indicated with stars, and 5’tRNA fragments are indicated with arrows. Statistical tests: One-tailed Student’s t-test (**C-F**). *p < 0.05.

**Additional file 5: Supplementary Figure S5.** 5’TOGs bind to translation initiation factors. **A)** No differences in protein expression of translation initiation complex factors were detected by western blotting in PC3 parental, WT, and *METTL1 KO* cells. The bottom boxplot shows the normalised protein level quantification, and the dotted lines indicate the mean protein levels in WT cells. **B)** Global protein synthesis rate measured by OP-puromycin (OP-puro) incorporation in PC3 *METTL1 KO* cells compared to the control (WT) after NaAsO_2_ exposure at the indicated time points. Fluorescence was normalised to cell size (FSC). *n* = 3; mean ± SD. **C)** Representative western blot showing translation initiation factors pulled down by m^7^G-cap sepharose beads in PC3 *METTL1 KO* vs. WT cell extracts. **D)** Representative western blot showing translation initiation factors pulled with synthetic biotinylated-5'TOG in PC3 WT cells transfected with biotinylated-5'TOGs (5'TOG), anti-TOG (ANT) or scramble RNA (-). **E)** Representative western blot showing mRNA-cap-bound translation initiation factors after 5'TOG (TOG), anti-TOG (ANT), or scramble control (Ctrl) transfection in PC3 WT and *METTL1 KO* cells. Quantification of western blots are shown in Fig. 5 (**C, D, E**). Statistical tests: One-tailed Student’s t-test (**A**), and multiple t-test (**B**); ***p < 0.001,

**Additional file 6: Supplementary Figure S6.** METTL1 mediated methylation promotes cell proliferation, and tumour growth in vivo. **A)** Proliferation of DU145 *METTL1 KO* and WT cell clones. Mean ± SD, *n* = 6. The thick dotted line represents the average growth of the WT and KO cells. **B)** Proliferation of *METTL1*-silenced 22Rv1 cells. Mean ± SD, *n* = 3. **C)** Cell cycle analysis of PC3 *METTL1 KO*, WT, and parental cells showing decreased cell cycle progression in *METTL1 KO* cells. Mean ± SD, *n* = 3. The dotted lines indicate the mean of three biological replicates. **D)** Representative images of BrdU staining of three clones of PC3 *METTL1 KO* and WT cells. Quantification is shown in Fig. 5. **E)** Schematic representation of generation of single-cell-derived spheroids, and representative images of single-cell-derived spheroids (lower panel). Quantification is shown in Fig. 5. **F)** *METTL1* depletion affects tumour growth in vivo. Immunohistochemical staining for METTL1, Ki67, and cleaved caspase 3 (Cl-Casp3) in PC3 *METTL1 KO* and WT cell-derived xenografts. Right charts show the quantification of Ki67 + and Cl-Casp3 + cells per microscopic field. Mean ± SD, *n* = 5, and ten images per biological replicate. **G-I**) Protein expression (**G**) and m^7^G methylation levels (**H, I**) of a second PC3 *METTL1 KO* cell clone (KO2) ectopically expressing an HA-tagged wild-type (WT) or catalytic dead mutant (AFPA) version of METTL1. *METTL1 KO2* cells were infected with the empty vector (eV) as a control. Methylene blue staining was used as the loading control (**H**, bottom panel). Mean ± SD, *n* = 3 (**H**). **J, K)** Proliferation (**J**) and spheroid formation capacity (**K**) of PC3 *METTL1 KO2* cells re-expressing METTL1 (WT) or catalytic dead mutant (AFPA) compared with *METTL1 KO2* (eV). Mean ± SD, *n* = 6. **L**) *WDR4* mRNA expression and cell growth of PC3 cells expressing doxycycline-inducible SCR shRNA or two shRNA against *WDR4* with or without doxycycline induction. Mean ± SD are represented (*n* = 6).** M**) *WDR4* mRNA expression and tumour growth of xenografts of cells expressing doxycycline-inducible SCR shRNA or a shRNA against *WDR4* with or without doxycycline induction. Mean ± SEM are represented (*n* = 10 for shWD2, *n* = 5 for SCR, for each Dox condition). Scale bar represents 100 μm (**D**), 50 μm (**F**). Statistical tests: Two-way ANOVA (**A, I, J**), one-way ANOVA (**C**), and one-tailed Student’s t-test (**B, F, K, L, M**). **p < 0.01, ***p < 0.001, ****p < 0.0001.

**Additional file 7: Supplementary Figure S7.** Translational and transcriptional characterisation of *METTL1 KO* cells. **A)** Differential mRNA expression ranked in a volcano plot according to their statistical *P*-value (-Log_10_ pV) and their relative abundance ratio (Log_2_ FC) between PC3 WT and *METTL1 KO* cells. Coloured dots represent statistically significant (*p* < 0.05) upregulated (red) and downregulated (blue) mRNAs levels. *n* = 3. **B)** *GAPDH* and *KIF20A* were not differentially translated in PC3 *METTL1 KO* cells. Percentage per fraction of mRNA abundance of the indicated genes found in polysomes of WT and *METTL1 KO* cell lysates. *n* = 3, mean ± SEM. The boxplot shows the fold change (FC) of mRNA abundance in polysome fractions of *METTL1 KO* versus WT cells. **C)** Global protein expression differences ranked in a volcano plot of PC3 *METTL1 KO* versus WT cells. **D)** Gene Ontology (GO) category enrichment of biological processes in significantly (*p* < 0.05) upregulated (UP) or downregulated (Down) proteins identified in PC3 *METTL1 KO* cells compared with WT cells. **E)** Western blots of PC3 WT and *METTL1 KO* cells transfected with synthetic 5’TOG RNAs (TOG), synthetic anti-TOG RNAs (ANT), or scramble RNAs (Ct). High (h) and low (l) western blot exposures are shown for pSTAT1, total STAT1 and IRF9. The bottom bar plots indicate the expression of each protein relative to GAPDH. Mean ± SEM, *n* = 3. **F)** Immunohistochemical staining for pSTAT1 in PC3 *METTL1 KO* and WT cell-derived xenografts. Scale bar represents 50 μm. **G, H)** Increased mRNA expression levels of ISGs genes in *METTL1* KO PC3 (**G**) and *METTL1*-silenced 22Rv1 cells (**H**) cells. Mean ± SD, *n* = 3. **I)** Western blot of IRF9 and METTL1 protein levels in *METTL1*-silenced 22Rv1 cells compared to control cells (SCR). Bottom bar plots show quantification of IRF9 expression normalised to GAPDH. Mean ± SD, *n* = 3. **J)** Correlation analysis between *METTL1* expression and ISG genes in three human PCa expression datasets reflects an inverse expression correlation. Correlations with *p*-value <  = 0.05 and |R|> = 0.2 are indicated with (*). Statistical tests: One-tailed (**B, G-I**) and two-tailed Student’s t-test and Pearson correlation test (**J**) were performed. *p < 0.05, **p < 0.01, ***p < 0.001, ****p < 0.0001.

**Additional file 8: Supplementary Figure S8.** Immune cell characterisation and correlation with *METTL1* expression in human PCa. **A)** Complete cytokine array analysis of PC3 *METTL1 KO* compared to WT cells’ c.m. shows differential expression of inflammatory cytokines. Mean ± SD of log_2_ fold change, *n* = 4. **B)** tSNE analysis of the expression markers of M1-, M2-like and Mø macrophages exposed to PC3 *METTL1 KO* and WT cells’ c.m. of a second biological replicate (KO2 and WT2). **C)** Phagocytic capacity of macrophages exposed to PC3 *METTL1 KO* and WT cells’ c.m. Mean ± SEM, *n* = 3. **D)** Polarisation of primary peripheral blood monocytes (PMBCs) exposed to PC3 *METTL1 KO* and WT cells’ c.m*.* The upper panel shows a schematic of the workflow used. The lower panels show the quantification of the percentage of positive cells. *n* = 3 technical replicates of 2 biological replicates. **E)** Activation of T cells from a second blood donor and co-culture with macrophages exposed to PC3 WT and *METTL1 KO* cells’ c.m. T cells were stained with CFSE on day 0 and measured on day 3. Means ± SD, *n* = 3 for two biological replicates. **F)** Representative immunohistochemical staining for METTL1 and CD86 (M1-like macrophages), CD163 (M2-likemacrophages), and CD8 T cells in human PCa biopsies. The scale bar represents 50 μm. **G, H)** *METTL1* mRNA expression inversely correlates with M1-like macrophage infiltration using the CIBERSORT deconvolution algorithm with TCGA and Taylor datasets (**G**), and TAM infiltration analysed using TIMER (**H**). Statistical tests: One-tailed t-test: *p < 0.05, **p < 0.01, ***p < 0.001 (**D, E**), Pearson’s correlation (r), *p*-value (pV), and linear regression with 95% confidence (bands) are shown (**G, H**).

**Additional file 9: Supplementary Figure S9. **Characterization of *Mettl1* deletion in murine PCa. **A)** Schematic overview of *Mettl1* systemic knockout and *Mettl1*^*flox/flox*^ mouse model generation. **B)** mRNA levels of *Mettl1* in prostatic tissue from five-month-old *Pten-KO/Mettl1+/+, Pten-KO/Mettl1+/-,* and *Pten-KO/Mettl1*^*fl/fl*^ mice. Mean ± SD, *n*=3. **C)** 5’tRNA fragment accumulation in *Pten-KO/Mettl1+/+, Pten-KO/Mettl1+/-* and *Pten-KO/Mettl1*^*fl/fl*^ tumours. The right bar chart shows means ± SD, n ≥5.** D)** Haematoxylin and eosin staining of *Pten-KO/Mettl1+/+, Pten-KO/Mettl1+/-,* and *Pten-KO/Mettl1*^*fl/fl*^ tumour sections from the ventral, dorsal, and anterior lobes. **E)** Representative images of immunostained sections for METTL1 and markers of proliferation (Ki67), M1-like (iNOS) and M2-like (CD68, Arg1) macrophages, and CD8+ T cells (CD8) from *Pten-KO/Mettl1+/+, Pten-KO/Mettl1+/-,* and *Pten-KO/Mettl1*^*fl/fl*^ prostate tumours*.* Arrows indicate positive cells. **F)** Prostate tumour volume from *Pten-KO/Mettl1+/+* and *Pten-KO/Mettl1+/-* five-month-old mice reflects reduced tumour burden after *Mettl1* deletion*.* Ventral (V), dorsal (D) and anterior (A) lobes. Mean ± SD, n≥3. **G)** Systemic deletion of *Mettl1* resulted in reduced tumour proliferation (Ki67+ cells) and increased immune infiltration of iNOS+ (M1-like) macrophages and CD8+ T cells. Staining of tumours from *Pten-KO/Mettl1+/+* and *Pten-KO/Mettl1+/-* five-month-old mice*.* Mean ± SD, n≥3, >10 images per biological replicate. **H)** Tumour growth of allografts derived from *Pten-KO/Mettl1+/+* and *Pten-KO/Mettl1*^*flox/flox*^ tumour cells injected into NSG male mice reflects not significant tumour formation reduction in the absence of *Mettl1*. Mean ± SEM, *n*=9. **I)** Prostate lobe volumes in *Pten-KO/Mettl1*^*flox/flox*^ (fl/fl) mice and *Pten-KO/Mettl1+/+* (+/+) mice treated with anti-CD8 antibodies or IgG control. Mean ± SD, (n≥6). **J)** Prostate lobe volumes in *Pten-KO/Mettl1*^*flox/flox*^ (fl/fl) mice and *Pten-KO/Mettl1+/+* (+/+) mice treated with anti-PD1+anti-CTLA4 antibodies or IgG control. Normal prostate lobe volumes (from wild-type mice) are also shown. The bar chart on the left shows the volume in separated lobes. The bar chart on the right shows the sum of the volume of all lobes. Mean ± SD, (fl/fl and +/+ n≥6; Normal *n*=5). Scale bars represent 10 mm (**D**), 50 μm (**E**). Statistical tests: One-tailed Student’s t-test (**G**), Mann-Whitney test (**C, F, H, I, J**), *p<0.05, **p<0.01, ***p<0.001.

**Additional file 10: Supplementary Table S1. **Differential expression of RNA modifying proteins in human Pca.

**Additional file 11: Supplementary Table S2. **METTL1 bound RNAs in PC3 cells.

**Additional file 12: Supplementary Table S3. **METTL1 bound RNAs in HEK293T cells.

**Additional file 13: Supplementary Table S4. **tRNA fragment reads in Control (wt) and METTL1 KO cells.

**Additional file 14: Supplementary Table S5. **Differentially synthesised nascent peptides in WT and METTL1 KO PC3 cells.

**Additional file 15: Supplementary Table S6. **Differentially expressed proteins in WT and METTL1 KO PC3 cells.

**Additional file 16: Supplementary Table S7. **Differentially expressed mRNAs in WT vs METTL1 KO PC3 cells.

**Additional file 17: Supplementary Table S8. **Correlation of differentially expressed mRNAs and newly synthesized proteins in WT and METTL1 KO PC3 cells.

**Additional file 18. **Graphical abstract.

## Data Availability

The transcriptomic data, PAR-CLIP and microarray generated in this publication have been deposited in NCBI’s Gene Expression Omnibus (GEO Series accession numbers GSE189883, GSE190911, and GSE230694) and the European Nucleotide Archive (ENA projects PRJEB49285 and PRJEB53441). The mass spectrometry proteomics data have been deposited to the ProteomeXchange Consortium via the PRIDE partner repository with the dataset identifier PXD030141.

## Abbreviations

3'tRF: 3'-Derived tRFs
5'TOGs: 5'-Terminal oligoguanine-containing tRNA fragments
5'tRF: 5'-Derived tRFs
AKT: AKT Serine/Threonine Kinase
anti-TOGs: Reverse-complement 5'TOG RNA
AR: Androgen receptor
BPH: Benign prostatic hyperplasia
c.m.: Conditioned medium
CRISPR: Clustered regularly Interspaced Short Palindromic Repeats
CRPC: Castration resistant prostate cancer
CTLA-4: Cytotoxic T-Lymphocyte Antigen 4
DF: Disease free
DFS: Disease free survival
DHT: Dihydrotestosterone
eIF2α: Eukaryotic initiation factor 2 alpha
eIF4A/G/E: Eukaryotic initiation factor-4A/G/E
FC: Fold change
FSC: Forward scatter
GAPDH: Glyceraldehyde-3-phosphate dehydrogenase
GM-CSF: Granulocyte–macrophage colony-stimulating factor
GO: Gene ontology
ICB: Immune checkpoint blockade
IFI44: Interferon induced protein 44
IFIT1: Signal transducer and activator of Transcription 1
IFIT2: Signal transducer and activator of Transcription 2
IFN: Interferon
IL3/10: Interleukin 3/10
int-tRF: Internal-derived tRFs
IRF9: Interferon regulatory factor 9
ISG15: Interferon-stimulated gene 15
ISGs: Interferon stimulated genes
KIF20A: Kinesin-like protein 20A
KLK3: Kallikrein-3
LC–MS-MS: Liquid Chromatography with tandem mass spectrometry
lincRNAs: Long intergenic noncoding RNAs
m^7^G: N^7^-methylguanosine
M-CSF: Macrophage or Colony stimulating factor
METTL1: Methyltransferase 1
miRNA: MicroRNAs
mRNA: Messenger RNA
mTOR: Mechanistic target of Rapamycin Kinase
ncRNAs: Noncoding RNAs
NK: Natural killer
NSG: NOD scid gamma
OP-puro: O-propargyl-puromycin
PABP1: Polyadenylate-binding protein 1
PAR-CLIP: Photoactivatable-Ribonucleoside-Enhanced cross-linking immunoprecipitation
PCa: Prostate Cancer
PD1: Programmed cell Death protein 1
PI3K: Phosphoinositide 3-kinase
PIN: Prostate intraepithelial neoplasia
PTEN: Phosphatase and tensin homolog
pV: *P* Value
RMP: RNA modifying proteins
S6K: Ribosomal protein S6 kinase
SD: Standard deviation
SEM: Standard error mean
scRNA-seq: Single-cell RNA sequencing
STAT1: Signal transducer and activator of transcription 1
TAM: Tumour associated macrophages
TCGA: The Cancer Genome Atlas
TIMER: Tumor Immune Estimation Resource
TME: Tumour microenvironment
TNF-α: Tumour necrosis factor α
tRFs: tRNA fragments
tRNA: Transfer RNA
tSNE: T-distributed Stochastic Neighbor Embedding
WDR4: WD Repeat Domain 4
YB1: Y-box transcription factor 1
